# Melatonin: A Potential Therapy for Osteoporosis With Insights Into Molecular Mechanisms

**DOI:** 10.1111/jpi.70062

**Published:** 2025-06-01

**Authors:** Ko‐Hsiu Lu, Yi‐Hsien Hsieh, Renn‐Chia Lin, Meng‐Ying Tsai, Shun‐Fa Yang

**Affiliations:** ^1^ Department of Orthopedics Chung Shan Medical University Hospital Taichung Taiwan; ^2^ School of Medicine Chung Shan Medical University Taichung Taiwan; ^3^ Institute of Medicine Chung Shan Medical University Taichung Taiwan; ^4^ Department of Medical Research Chung Shan Medical University Hospital Taichung Taiwan

**Keywords:** mechanism, melatonin, osteoporosis, therapy

## Abstract

Melatonin is a versatile neurohormone with diverse molecular functions, including sleep regulation, inflammation reduction, antioxidant activity, immune modulation, and anticancer properties. In bone metabolism, it promotes osteoblast formation, inhibits osteoclast activity, and synchronizes skeletal tissue rhythms to support bone health. As melatonin is not yet clinically used for osteoporosis and concerns about the current treatments' side effects remain, this review highlights its role in modulating osteoblast and osteoclast interactions, particularly through regulation of the receptor activator of nuclear factor‐κB ligand and osteoprotegerin, to achieve bone‐forming and antiresorptive effects. These effects have been demonstrated across various concentrations in diverse cell types and In Vivo models. Furthermore, melatonin safeguards the bone microenvironment by mitigating oxidative stress and inflammation, protecting osteoblasts, preventing bone loss, and maintaining the gut microbiota and brain–gut–bone axis. These attributes underscore melatonin's potential as an effective alternative or complementary therapy for promoting bone health and managing osteoporosis. Future research is needed to determine optimal dosing and timing for maximum efficacy.

## 1 Introduction

Melatonin (*N*‐acetyl‐5‐methoxytryptamine, C_13_H_16_N_2_O_2_) is a hormone primarily produced by the pineal gland in response to darkness, regulating the circadian cycle and being inhibited by light exposure [1]. Its nocturnal secretion peaks at night, influencing the sleep–wake cycle, pubertal development, immune responses, bone homeostasis, and seasonal adaptation [2]. Melatonin production is directly linked to night length and synthesized from tryptophan, exhibiting both chronobiotic and cytoprotective properties [2, 3]. Pineal‐derived melatonin coordinates circadian rhythms across various tissues, including bone, where it contributes to metabolism and growth [4]. Bone cells operate within a 24‐h cycle, influenced by factors such as age, gender, hormonal status, and pathological conditions [2]. The circadian oscillator system comprises a complex network of central and peripheral clocks, harmonized by melatonin from both pineal and extra‐pineal sources [2, 5].

Extra‐pineal melatonin, such as found in the gastrointestinal tract, supports mucosal integrity through its antioxidant and free radical‐scavenging properties [5]. In the bone marrow and thymus, melatonin modulates immune function and exhibits cytoprotective effects, including neuroprotective, anti‐inflammatory, antioxidant, and antitumor activities [6]. Melatonin concentrations in bone marrow surpass those in nocturnal blood, influencing the differentiation of osteoclast and osteoblast while acting as an autacoid in bone tissue. Through interactions with melatonin receptors (MRs) and its receptor‐independent antioxidant mechanisms, melatonin regulates bone turnover, which aligns with the circadian rhythm [7]. Age‐related decline in melatonin, particularly pronounced in postmenopausal women, is associated with increased nighttime bone resorption, thereby accelerating osteoporosis and elevating fracture risk [8]. Additionally, melatonin has been linked to bone‐related disorders such as osteoarthritis and osteosarcoma [1, 2].

## 2 Osteoporosis

Osteoporosis, the most prevalent bone disease in older adults, is characterized by bone loss, increased fracture risk, and significant health complications, particularly affecting the hip and spine [9, 10]. It results from disrupted bone remodeling, where heightened osteoclast activity and diminished osteoblast function create an imbalance that favors bone resorption. Primary osteoporosis includes two major types: postmenopausal osteoporosis, driven by estrogen deficiency, and senile osteoporosis, linked to aging [10]. Secondary osteoporosis, on the other hand, arises from underlying medical conditions such as chronic kidney disease, hyperthyroidism, hypogonadism, malabsorption syndromes, and rheumatoid arthritis, or the use of certain medications such as anticonvulsants, aromatase inhibitors, glucocorticoids, proton pump inhibitors, and selective serotonin reuptake inhibitors [11]. Diagnosis is primarily based on bone mineral density (BMD) assessment using central dual‐energy X‐ray absorptiometry (DXA) [12]. The World Health Organization (WHO) defines osteoporosis as a BMD *T*‐score of −2.5 or lower, with osteopenia, a precursor to osteoporosis, classified by a BMD *T*‐score ranging between −1.0 and −2.5.

### 2.1 Skeletal System

The skeleton is composed of two primary tissues: bone and cartilage. Bone tissue contains osteoblasts (bone‐forming cells) and osteoclasts (bone‐resorbing cells), while chondrocytes populate cartilage [13]. Bone cells regulate and shape bones through modeling and remodeling processes. Bone marrow mesenchymal stem cells (BMSCs) can differentiate into osteoblasts, chondrocytes, and adipocytes [14]. Osteoblasts, derived from MSCs, drive bone formation and mineralization via endochondral ossification (mediated by chondrocytes) or intramembranous ossification [15]. Osteoclasts, large multinucleated cells derived from hematopoietic monocytes or macrophages, are regulated by macrophage‐colony stimulating factor (M‐CSF) and the receptor activator of nuclear factor‐κ‐light‐chain enhancer of activated B cells (NF‐κB) ligand (RANKL) [7, 15]. Bone turnover is influenced by neural and hormonal signals, including melatonin from the pineal gland and bone marrow, which exert antioxidative and immunomodulatory effects [7, 15].

### 2.2 Current Treatment

Postmenopausal osteoporosis is primarily driven by increased bone resorption caused by estrogen deficiency [10]. Hormone replacement therapy can lower bone resorption to premenopausal levels but carries substantial risks, including cancer, cardiovascular disease, and thromboembolism [9, 10]. Current treatments focus on enhancing osteoblast activity, reducing bone resorption, or addressing both processes. Bone‐forming therapies stimulate bone formation, such as teriparatide, abaloparatide, full‐length parathyroid hormone (PTH), and romosozumab [9, 10]. However, most available treatments target osteoclast inhibition to prevent further bone loss, often without substantially increasing bone mass.

Anti‐resorptive drugs, including bisphosphonates (BPs), denosumab, raloxifene, and calcitonin, are widely recognized for their ability to improve BMD and reduce fracture risk. However, their clinical use is often constrained by notable side effects, such as osteonecrosis of the jaw, atypical fractures, rebound vertebral fractures, fetal stroke, and venous thromboembolism [9, 16]. Additionally, limitations in long‐term efficacy and tolerability contribute to suboptimal patient adherence [9, 16, 17]. This review explores the potential of melatonin as a promising adjuvant therapy for osteoporosis. Melatonin enhances osteoblast differentiation, inhibits osteoclast activity, and exhibits dose‐dependent anti‐osteoporotic effects, ranging from physiological concentrations (nM) to pharmacological (µM–mM) levels [18, 19, 20]. These effects vary across In Vitro and In Vivo models [18, 19, 20]. Accordingly, we focus on the impact of melatonin at different doses in diverse cellular and animal studies to elucidate its mechanisms and efficacy in promoting bone health and managing osteoporosis.

## 3 Melatonin

In adults, daytime blood melatonin levels average around 10 pg/mL, rising to approximately 60 pg/mL at night. These levels begin to elevate around 10 PM, peak near 2 a.m., and remain elevated for about 9 h, following the natural light/dark cycle [21]. Melatonin secretion peaks in prepubertal children aged 4–7 years, with morning serum levels consistently below 20 pg/mL across all ages [14]. Nighttime melatonin levels are initially low during the first 6 months of life (27.3 ± 5.4 pg/mL) but increase to a peak between 1 and 3 years (329.5 ± 42.0 pg/mL). They then gradually decline with age: from 210 ± 35 pg/mL in early childhood (1–5 years) to 133 ± 17 pg/mL at 5–11 years. During adolescence (15–20 years), levels decrease further to 62.5 ± 9.0 pg/mL, and in young adulthood, they fall to 46 ± 6 pg/mL, representing a 75% reduction from peak childhood levels [21, 22]. This decline continues into old age (70–90 years), with levels dropping to 29.2 ± 6.1 pg/mL, approximately 20% of the peak levels observed in early childhood. Bone marrow melatonin levels decrease with age, dropping from approximately 400 pg/mL in individuals under 45 to less than 250 pg/mL in those over 60 [23]. Melatonin levels also decline significantly during menopause and with prolonged immobility, although exercise has been shown to increase them [8, 24, 25]. Circadian disruptions caused by aging, excessive light exposure, or shift work are associated with greater bone loss and an elevated risk of fractures [26]. Bone turnover exhibits a 24‐h rhythm, and low melatonin levels, particularly in postmenopausal obese women, accelerate type I collagen (COL1) turnover and bone loss [27]. Melatonin regulates the human menstrual cycle, rising in the late luteal phase likely due to progesterone and declining from premenopause to postmenopause [28]. During adolescence, melatonin is vital for bone metabolism, and its disruptions are linked to idiopathic adolescent scoliosis [29]. With aging, melatonin levels enhance bone resorption and loss, reducing BMD and the development of postmenopausal and senile osteoporosis [30].

### 3.1 Biosynthesis and Metabolism

Tryptophan, the precursor to melatonin, is absorbed from the bloodstream and converted into 5‐hydroxytryptophan by the enzyme tryptophan‐5‐hydroxylase [14]. Next, 5‐hydroxytryptophan is decarboxylated by 5‐hydroxy‐l‐tryptophan decarboxylase to form serotonin. Serotonin is then acetylated by bisphosphonate (AANAT), resulting in the formation of *N*‐acetylserotonin. Finally, *N*‐acetylserotonin is methylated by hydroxyindole‐*O*‐methyltransferase (HIOMT), also known as *N*‐acetylserotonin methyltransferase (ASMT), to produce melatonin [1]. Once synthesized, melatonin is released into capillaries and cerebrospinal fluid, where it is rapidly distributed throughout body tissues.

Over 90% of circulating melatonin is metabolized in the liver, primarily by cytochrome P450 enzymes, mainly CYP1A2 and CYP1A1 [14]. These enzymes convert melatonin into 6‐hydroxymelatonin, which is then conjugated with sulfate to form 6‐sulfatoxymelatonin and excreted in bile. This process results in rapid first‐pass metabolism and a short half‐life of 30–60 min. A smaller fraction of melatonin undergoes *O*‐demethylation to produce *N*‐acetyl‐5‐hydrotryptamine, which is conjugated with sulfuric acid (90%) or glucuronic acid (10%) and eliminated via urine. Approximately 5% of circulating melatonin is excreted unchanged. A minor metabolic pathway involves the deacetylation of melatonin to form 5‐methoxytryptamine, which can give rise to compounds such as pinoline, bufotenine, *N*,*N*‐dimethyltryptamine, and 5‐hydroxytryptamine, potentially converting back into melatonin [31].

After oral ingestion of 80 mg of melatonin by young male volunteers, peak plasma levels are reached 60–150 min later, with concentrations 350–10,000 times higher than physiological levels at nighttime [30, 32, 33], while its bioavailability remains low (~15%) after 2 and 4 mg oral dosage due to first‐pass metabolism [30, 32, 33]. Lower oral doses of melatonin (0.2–0.4 mg) produce plasma concentrations approximately 200–400 pg/mL, considered physiological in healthy volunteers [32]. With immediate‐release formulations, the time to peak concentration is ~50 min, and the half‐life is ~45 min for both oral and intravenous administration. Thus, melatonin should be taken 45 min before the desired effect [32].

Intravenous administration bypasses the first‐pass effect, yielding a 2‐min distribution half‐life and a 20‐min metabolic half‐life [14]. At pharmacological doses, the liver's capacity to metabolize melatonin may be surpassed, leading to an increased excretion of unchanged melatonin and systemic exposure [30]. These pharmacokinetics influence melatonin's bone‐protective effects, as its bioavailability and systemic levels regulate osteoblast and osteoclast activity. Melatonin promotes osteoblast differentiation and mineralization via Runx2 signaling, suppresses osteoclast‐mediated bone resorption by modulating the RANKL/osteoprotegerin (OPG) ratio, and improves bone mass, microstructure, and biomechanical properties [7, 34]. In preclinical models, these concentrations enhance osteoblast differentiation and activity and reduce osteoclastogenesis via RANKL, promoting bone formation and increasing bone mass [7, 35, 36, 37]. Clinical evidence suggests potential benefits for bone turnover and BMD with efficacy dose‐dependent in postmenopausal women, although optimal dosing strategies require further investigation [38]. Excessive levels may cause receptor desensitization, while intravenous administration shows promise for acute bone loss, highlighting the need to refine dosing strategies in osteoporosis treatment [38].

### 3.2 MRs

Melatonin affects bone cells through three main mechanisms: binding to plasma membrane receptors (MT_1_ and MT_2_), interacting with intracellular proteins, like calmodulin and tubulin, and engaging nuclear orphan receptors such as the retinoid Z receptor/retinoid orphan receptor α (RZR/RORα). The MT_1_ and MT_2_ receptors, which are part of the G protein‐coupled receptor family, exert different effects on bone cells based on their location and activation [39, 40]. In blood vessels, activation of the MT_1_ receptor leads to vasoconstriction, while MT_2_ receptor activation causes vasodilation [39]. In bone tissue, MT2 is the primary receptor regulating bone formation and osteoclast inhibition, as well as modulating OPG/RANKL balance, NF‐κB inactivation, and cortical bone formation [7, 36, 41, 42, 43, 44, 45]. It exerts these effects through multiple signaling pathways, including MT2/Gi/β‐arrestin/mitogen‐activated protein kinase (MAPK)/extracellular signal‐regulated kinase (ERK) kinase (MEK)/ERK1/2, MT2/MEK/ERK5, and MT2/NF‐κB [7, 36, 41, 42, 43, 44, 45].

Both MT_1_ and MT_2_ receptors guide BMSCs toward differentiation into osteoblasts rather than adipocytes [46]. Melatonin regulates bone mass and the differentiation of MSCs into osteogenic, chondrogenic, adipogenic, or myogenic lineages through both MT_2_‐dependent and receptor‐independent pathways [43]. It also crosses cell membranes to bind to cytoplasmic proteins, such as quinone reductase, tubulin, and calmodulin, scavenging free radicals and influencing calcium signaling via adenylate cyclase and phosphodiesterase [39]. In the nucleus, melatonin activates silent information regulator type 1 (Sirtuin 1, SIRT1) and interacts with RZR/RORs [47].

### 3.3 Melatonin Receptor‐Dependent and Independent

Melatonin regulates bone remodeling via receptor‐dependent and receptor‐independent mechanisms, as well as its role in circadian regulation [48]. Through MT_1_ and MT_2_ receptors, melatonin enhances antioxidant enzyme activity, decreases pro‐oxidant enzyme levels, and reduces cytokine production, with MT_2_ being particularly critical for bone health [41]. In osteoblasts and MSCs, melatonin promotes osteoblastogenesis by activating MT_2_ receptors and triggering pathways such as MAPK, NF‐κB, and β‐arrestin [7]. It also suppresses osteoclastogenesis by reducing RANKL secretion [7, 45]. Furthermore, melatonin binds to calmodulin, influencing nitric oxide (NO) production, and may act on nuclear receptors such as RZR/RORα [49]. Inactivation of RORα decreases inhibitor of κB (IκB) expression, thereby activating NF‐κB and promoting antioxidant enzyme expression, while melatonin nonreceptor‐mediatedly scavenges reactive oxygen and nitrogen species (ROS/RNS) to protect mitochondrial proteins and DNA [49]. By modulating the ubiquitin‐proteasome system, melatonin impacts protein degradation and inhibits Ca^2+^/calmodulin‐dependent kinase II through its interaction with calmodulin, potentially regulating clock genes via proteasome inhibition [50]. It also enhances late‐stage osteoblast differentiation by upregulating and stabilizing osterix expression, thereby promoting osteogenic activity and bone mineralization, and highlighting its potential as a therapeutic agent for osteoporosis [51]. At physiological concentrations, melatonin regulates various physiological processes through cell surface receptors MT_1_ and MT_2_ [7, 36, 42, 43, 44, 45, 52]. However, at pharmacologically higher concentrations, such as 500 µM in mouse bone marrow macrophages (BMMs) [52], it inhibits osteoclast differentiation independently of MT receptors and directly functions as an intracellular antioxidant to scavenge free radicals (redox regulation) [49, 50].

## 4 Mechanisms of Melatonin on Bone Metabolism

The transforming growth factor‐β (TGF‐β) superfamily, including TGF‐βs, bone morphogenetic proteins (BMPs), and activins and related proteins, promotes osteoblastogenesis by binding to receptors on osteoblasts and activating signaling cascades that phosphorylate suppressor of mothers against decapentaplegic (SMAD) proteins and MAPKs [53]. This activation stimulates key osteoblastic factors, including RUNX2, BMP‐2, and osterix [54]. Meanwhile, the NF‐κB pathway regulates osteoclast‐specific genes such as the osteoclast‐associated receptor (*Oscar*), nuclear factor of activated T cells, cytoplasmic 1 (*Nfatc1*), tartrate‐resistant acid phosphatase (*Trap*), and cathepsin K (*CatK*) [55].

**Figure 1:**
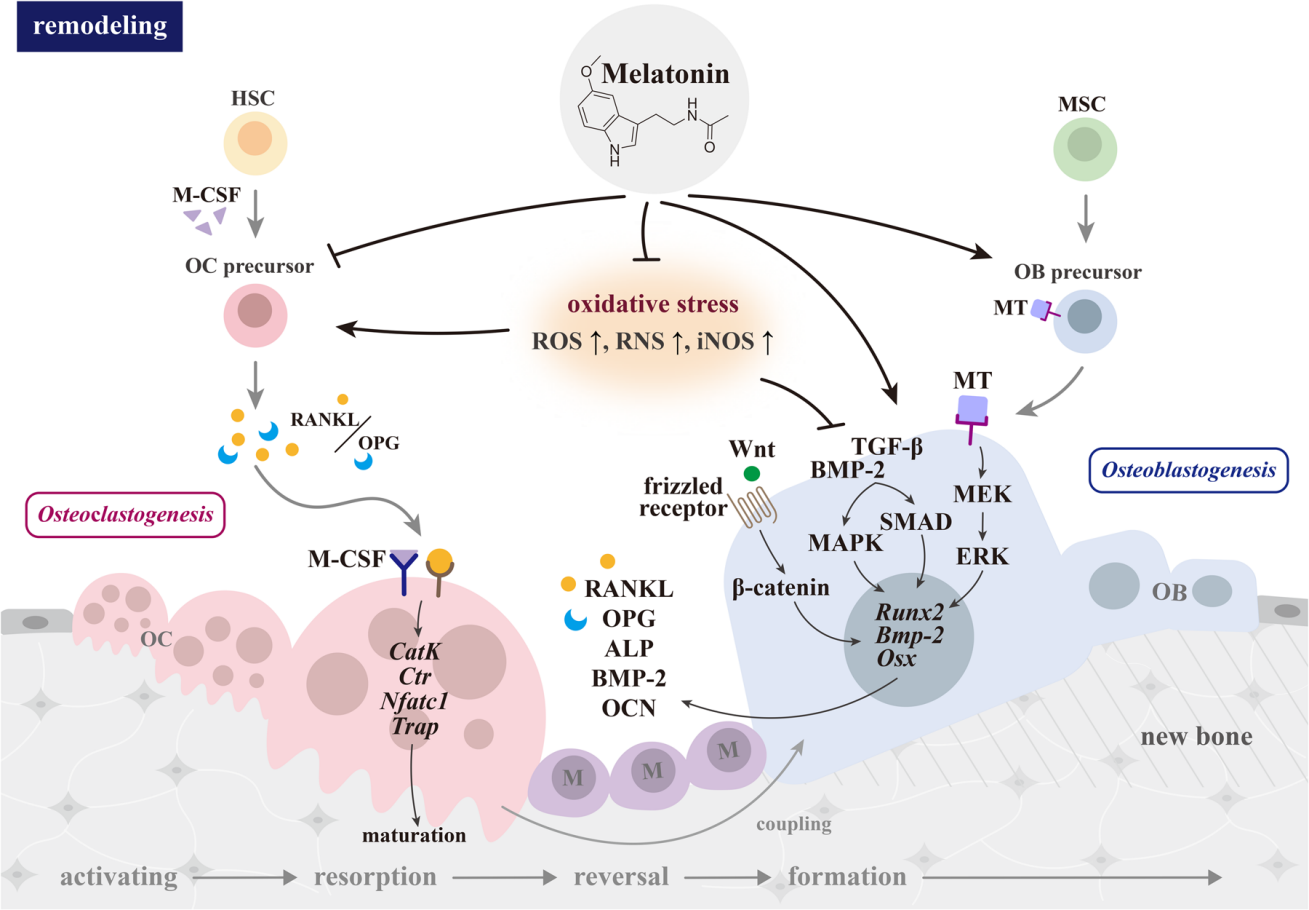
Effects of melatonin on bone remodeling and its regulation of osteoclasts and osteoblasts. Bone remodeling maintains adult skeletal health by coupling bone resorption by osteoclasts with bone formation by osteoblasts. In osteoporosis, bone resorption exceeds formation, resulting in bone loss. ALP: alkaline phosphatase; BMP‐2/*Bmp‐2*: bone morphogenetic protein 2/gene; Ca: calcium; *CatK*: cathepsin K gene; *Ctr*: calcitonin receptor gene; ERK: extracellular signal‐regulated kinase; HSC: hematopoietic stem cell; iNOS: inducible nitric oxide synthase; M: macrophage; MAPK: mitogen‐activated protein kinase; M‐CSF; macrophage‐colony stimulating factor; MEK: MAPK/ERK kinase; MSC: mesenchymal stem cell; MT: melatonin receptor; *Nfatc1*: nuclear factor of activated T cells gene, cytoplasmic 1; OB: osteoblast; OC: osteoclast; OPG: osteoprotegerin; *Osx*: osterix gene; RANKL: receptor activator of nuclear factor‐κB ligand; RNS; reactive nitrogen species; ROS: reactive oxygen species; *Runx*: runt‐related transcription factor gene; SMAD: suppressor of mothers against decapentaplegic; TGF‐β: transforming growth factor‐β; *Trap*: Wnt: wingless‐related integration site.

In the elderly, the bone resorption marker amino‐terminal cross‐linking telopeptide of COL1 (NTX‐1) exhibits diurnal variation, with women showing higher peaks due to increased bone resorption [56, 57]. Melatonin emerges as a promising agent for bone health because of its dual effects on osteoblasts and osteoclasts [58, 59] (Figure 1). It supports bone homeostasis by enhancing osteogenesis and reducing the RANKL/OPG ratio to inhibit osteoclast activity, oxidative stress, and inflammation [60].

### 4.1 Effects of Melatonin on Osteoblastogenesis

In mature female goldfish, melatonin at physiological to pharmacological levels (10 nM to 10 µM) inhibited both osteoblastic and osteoclastic activities in scales by reducing TRACP and alkaline phosphatase (ALP) activities and downregulating estrogen receptor (ER) and insulin‐like growth factor (IGF)‐1 mRNA expression [61] (Table 1). Similarly, in zebrafish scales, melatonin at 100 nM suppressed osteoblast and osteoclast differentiation by inhibiting epidermal ERK signaling pathways [93].

**Table 1:** Effects of melatonin on osteoblastogenesis.

Reference	Model	Dose and route	Effective dose	Evidence and mechanism
Roth JA, 1999 [35]	Mouse MC3T3‐E1 cell ROS17/2.8 cell	1–100 nM	50 nM	Promotes differentiation and mineralization of osteoblast growth
Nakade O, 1999 [62]	HOB‐M cell Human SV‐HFO cell	5–100 µM	5–100 µM	Increases bone cell proliferation and COL1 synthesis
Koyama H, 2002 [20]	Male *ddy* mouse	1, 5, and 50 mg/kg/day IP	5 and 50 mg/kg/day	Increases BMD by 36%, bone mass by 49%, and trabecular thickness by 19% via inhibition of bone resorption
	Mouse MC3T3‐E1 cell	5–500 µM	5–500 µM	Reduces RANKL mRNA and OPG mRNA and protein
Suzuki N, 2002 [61]	Female matured goldfish	1 nM to 10 µM	100 nM to 10 µM	Affects scale osteoblastic cells by inhibiting ALP via reduction of ER and IGF‐1 mRNAs
Oktem G, 2006 [63]	Female Wistar rat	10 mg/kg/day SC	10 mg/kg/day	Rescues iNOS increase, apoptotic cells in nucleus pulposus and epiphyseal cartilage, and osteoblasts decrease
Radio NM, 2006 [42]	Human MSC	1 fM to 1 µM	50 nM	Promotes ALP in osteogenesis via MT_2_, MEK/ERK1/2, EGFRs, metalloproteinase, and clathrin‐mediated endocytosis, but not PKA
Uslu S, 2007 [64]	Female Wistar rat	10 and 30 mg/kg/day SC	10 and 30 mg/kg/day	Reverses decreases of trabecular thickness, area of vertebra and femur, and cortical thickness of femur
Satomura K, 2007 [18]	Human osteoblastic cell	1–200 µM	100 µM	Enhances human osteoblastogenesis
	Male mouse	100 mg/kg IP	100 mg/kg	Promotes mouse cortical bone formation
Sanchez‐Hidalgo M, 2007 [65]	Rat ROS17/2.8 cell	1 nM to 1 mM	100 nM	Inhibits fatty acid‐induced triglyceride accumulation Impacts osteoblastogenesis and osteoporosis
Zaminy A, 2008 [66]	Rat BMSC/AMSC	20–200 pg/mL	20–200 pg/mL	Promotes osteogenesis in BMSCs but not in AMSCs.
Sethi S, 2010 [36]	Human AMSC	50 nM	50 nM	Activates osteoblastogenesis by forming the MT_2_R/Gi/β‐arrestin/MEK/ERK1/2 complexes to induce osteogenesis
Zhang L, 2010 [67]	Human MSC	10 nM to 100 µM	100 µM	Ameliorates adipogenesis and promotes osteogenesis by inhibiting PPARγ and activating RUNX2
Liu L, 2011 [68]	hFOB 1.19 cell	1 nM to 1 mM	1 mM	Delay proliferation by inducing G1 and G2/M arrest
Park KH, 2011 [69]	Mouse MC3T3‐E1 cell	12.5–100 nM	50 nM	Enhances osteoblastogenesis and mineralization via BMP/ERK/Wnt
William P, 2012 [70]	Mouse NIH3T3 fibroblast Human osteoblast hMSC	1 mg/mL	1 mg/mL	CA‐melatonin scaffolds enhance the adhesion, viability, and proliferation, osteoconductive and osteoinductive properties
Witt‐Enderby PA, 2012 [71]	MMTV*‐Neu* female transgenic mouse	0.05 mg/night oral	0.05 mg/night	Increases bone density by 20% and regulates osteoblastogenesis genes after 1 year
Kotlarczyk MP, 2012 [72]	Human (RCT)	3 mg/day	3 mg/day	Restore imbalances in bone remodeling to prevent bone loss
Kim CH, 2013 [58]	Mouse MC3T3‐E1 cell	0.1 and 1 mM	1 mM	Promotes osteoblast anabolic response by stimulating p‐ERK and MnSOD with fluid shear stress
Liu X, 2013 [115]	Human MSC	10 nM to 100 µM	1 and 100 µM	Improves proliferation, protects viability against IL‐1β, and reduces ROS by upregulating SOD expression
Zhang L, 2013 [73]	Human MSC	10 mg in PLGA (40% in the first 3 days)	10 mg	Increases ALP expression, activity, and osteoblastogenesis markers (RUNX2, OPN, and OCN)
Tresguerres IF, 2014 [34]	Male Wistar rat	10 mg/kg/day oral	10 mg/kg/day	Improves bone mass, microstructure, and biomechanical properties
Gao W, 2014 [52]	hMSC	50 nM	50 nM	Promotes chondrogenesis via MTs
Son JH, 2014 [111]	Mouse MC3T3‐E1 cell	10–250 µM	100 µM	Enhances osteoblastogenesis of MC3T3‐E1 cells under hypoxia via p38 and Prkd1
Satué M, 2015 [74]	Mouse MC3T3‐E1 cell	1 µM to 2.5 mM	2.5 mM	Decreases metabolic activity and total RNA content while raising cytotoxicity (2.5 mM) Decreases *Rankl* (0.01–1 mM), *Opg* (0.1 mM), and OCN release (0.1 mM) while increasing ERK1/2 (0.1 mM)
Xiong XC, 2015 [75]	hFOB 1.19 cell	1 nM to 100 µM	10 nM to 100 µM	Promotes osteoblast proliferation via c‐Raf, MEK1/2, ERK1/2, p90RSK, and MSK1
Amstrup AK, 2015 [38]	Human (RCT)	1 and 3 mg/day	1 and 3 mg/day	Increases BMD at the femoral neck dose‐dependently At high doses, increases vBMD in the spine
Zhou L, 2015 [76]	Human MSC	10 nM, 1 µM, and 100 µM	100 µM	Rescues H_2_O_2_‐induced premature senescence and osteogenesis potentials via TIRT1
Lian C, 2016 [117]	Human MSC	100 µM	100 µM	Reverses TNF‐α‐inhibited osteogenesis by reducing TNF‐α‐induced SMURF1 and ubiquitination and degradation of SMAD1
Zhang WL, 2016 [120]	hFOB1.19 cell	10 µM and 1 mM	10 µM and 1 mM	Promotes osteogenesis and inhibits autophagy in osteoblasts by inhibiting ERK (10 µM > 1 mM)
	Diabetic type 2 SD rat	50, 75, and 100 mg/kg IP	50, 100 mg/kg	Delays diabetes‐induced osteoporosis and reduces autophagy (50 mg/kg/day > 100 mg/kg)
Yildirimtur S, 2016 [77]	Diabetic type 1 SD male rat	250 µg/day IP	250 µg/day	Reduces oxidative stress biomarkers and improves bone healing in diabetic rats
Ping Z, 2017 [78]	Male C57BL/6J mouse calvarial model	5 and 50 mg/kg/day IP	5 and 50 mg/kg/day	Alleviates Ti‐induced suppression of osteogenesis and enhances new bone regeneration by increasing ALP, osterix, and OCN via Wnt/β‐catenin
	Mouse MSC	1 µM to 100 mM	1 µM to 100 mM	Alleviates Ti‐induced suppression of osteogenesis by increasing *Runx2*, *Osterix*, and *OCN* mRNA via Wnt/β‐catenin
Han Y, 2017 [51]	Mouse C2C12 cell	0.1–5 µM	1 µM	Regulates the later stage of osteoblastogenesis by enhancing osterix
Chu ZM, 2017 [79]	SD male rat	50 mg/kg/day IP	50 mg/kg/day	Promotes osteogenesis and bone formation Inhibits adipogenic differentiation
Maria S, 2017 [37]	Postmenopausal osteopenic women	5 mg (MSDK, In Vivo)	5 mg	Increases BMD and P1NP and decreases CTX/P1NP Improves mood and sleep quality
	Human MSC + human PBM	50 nM (MSDK, In Vitro)	50 nM	Increases in osteoblastogenesis and OPG, decreases in osteoclastogenesis and RANKL
	Human osteoblast			Increased pERK1/2 (MAPK1/MAPK3) and RUNX2 and decreased ERK5 (MAPK7), PPARγ (PPARG), and GLUT4 (SLC2A4)
	Human AMSC			Induced osteoblastogenesis
Sharan K, 2017 [43]	C3H/HeJ and mouse female C57Bl6	10 and 100 mg/kg/day oral	100 mg/kg/day	Enhances bone mass via MT_2_ Cures mice from OVX‐induced bone loss and restores bone quality
	Mouse calvarial osteoblasts	1, 10, and 100 nM	100 nM	Regulates osteoblast functions via MT_2_
Maria S, 2018 [7]	Human MSC and PBMC	50 nM	50 nM	Via MT_2_, MEK/ERK1/2, and MEK/ERK5: increases OPG/RANKL by inhibiting RANKL increases RUNX2 and NF‐κB decreases PPARγ, GLUT4, insulin Rβ, and integrin β1
	MMTV‐HER2/*neu* mouse	15 mg/mL/day oral	15 mg/mL/day	Increased OPG/RANKL (25 kDa) ratio, p‐ERK1/2, p‐ERK5, and RUNX2 in mouse tibia
	Human AMSC	50 nM	50 nM	Induces osteoblastogenesis similar to BMSCs
Kim CH, 2018 [80]	Mouse MC3T3‐E1 cell	0.1 and 1 mM	1 mM	Enhances ERK/Akt/mTOR signal in cilia‐less MC3T3‐E1, in combination with fluid shear stress
Palin LP, 2018 [81]	Wistar male rat	5 mg/kg/day oral	5 mg/kg/day	Restores bone repair by pinealectomy during osseointegration
Bae WJ, 2018 [82]	Human cementoblast and PDLC	10, 50, and 100 µM	100 µM	Blocks ethanol's premature senescence and osteoclastogenesis via activation of mTOR, AMPK, MAPK, and NFATc‐1 pathways, which was reversed by inhibition of PIN1
Lee S, 2018 [116]	hMSC	1–1000 µM	10, 100 µM	Promotes osteogenesis by activating AMPK and increasing FOXO3a and RUNX2
Dong P, 2018 [83]	Rat MSC	2 mM	2 mM	Increases NPY and NPY1R, promoting MSC proliferation, migration, and osteoblast differentiation
Tao L, 2018 [84]	hFOB 1.19 cell	1–4 mM	2 mM	Regulates CRE‐dependent gene transcription for osteoblast proliferation by activating Src and PKA
Xu L, 2018 [113]	Female C57BL/6J mouse	10 and 50 mg/kg/day IP	10 and 50 mg/kg/day	Attenuates OVX‐induced osteoporosis and impaired osteogenesis by inactivating NLRP3 inflammasome via Wnt/β‐catenin
	Mouse BMSC	50 and 100 µM	100 µM	Suppresses OVX‐induced NLRP3 and active IL‐1β, with DKK1 partially reversing its effects Inhibits NLRP3 inflammasome activation via Wnt/β‐catenin
Wu Z, 2018 [85]	hBMSC	50 nM	50 nM	Enhances chondrogenesis by upregulating miR‐526b‐3p and miR‐590‐5p to increase p‐SMAD1 via *Smad7*
Meng X, 2018 [86]	hFOB 1.19 cell	2, 4, and 6 mM	4 mM	miR‐590‐3p mediates melatonin‐induced cell apoptosis by targeting septin 7 in hFOB 1.19
Rafat A, 2019 [87]	Rat BMSC/AMSC	5 µM	5 µM	Preconditioning improves BMSCs and AMSCs survival, reduces apoptosis, and enhances osteogenesis
Zhou W, 2019 [88]	SD OVX rat	50 mg/kg/day IP	50 mg/kg/day	Increases bone mass by reducing mitochondrial oxidative stress via SIRT3/SOD2
	Mouse MC3T3‐E1 cell	100 µM	100 µM	Decreases H_2_O_2_‐induced oxidative stress and restores the osteogenesis potential
Qiu X, 2019 [89]	hBMSC	100 µM	100 µM	Counteracts TNF‐α‐induced ROS and osteogenesis inhibition Suppresses p‐p65 and blocks IκB‐α degradation, thus decreasing NF‐κB
Li Y, 2019 [90]	Female C57BL/6J mouse BMSC	1, 10, and 100 µM	10 µM	Increases miR‐92b‐5p to stimulate osteogenesis by targeting ICAM‐1
Li X, 2019 [59]	hBMSC Mouse MC3T3‐E1 cell	1 mM	1 mM	Enhances bone formation via canonical Wnt4‐Fzd1/6‐LRP5/6b‐catenin and noncanonical Wnt4‐Fzd2‐JNKp38 pathways Inhibits suppression of TNF‐α and NF‐κB on osteogenesis Wnt4‐dependently
Qiu S, 2020 [91]	Mouse MC3T3‐E1 cell	1, 2, and 4 mM	4 mM	Induces mitochondrial apoptosis in osteoblasts by upregulating STIM1, increasing cytosolic calcium and ERK
Zhao R, 2020 [92]	Mouse MC3T3‐E1 cell	1, 10, and 100 µM	1 µM	Rescues glucocorticoid‐induced inhibition of osteoblastogenesis via PI3K/AKT and BMP/SMAD
Kobayashi‐Sun J, 2020 [93]	Zebrafish scale	100 nM	100 nM	Suppresses both osteoblastogenesis and osteoclastogenesis via epidermal ERK inhibition
Ma H. 2020 [94]	Mouse MC3T3‐E1 cell	1, 10, and 100 µM	100 µM	Decreases ferroptosis and increases the osteogenic capacity via Nrf2/HO‐1
	Diabetic type 2 SD rat	10 and 50 mg/kg/day IP	10 and 50 mg/kg/day	Augments bone formation and mass in diabetic rats
Zhou Y, 2020 [45]	Female C57BL/6J mouse	10 and 100 mg/kg/day oral	10 and 100 mg/kg/day	Transforms the cytokine framework and improves bone mass in OVX mice
	Mouse BMSC	10 and 100 nM and 1–100 µM	10 and 100 nM	Increases osteogenic differentiation at 10 and 100 nM by inhibiting MT_2_‐dependent NF‐κB, but not on osteogenesis at 1–100 µM Decreases osteoclastogenesis by indirectly inhibiting RANKL
Murodumi H, 2020 [95]	hOB cell	0.2, 1, and 2 µM	1 µM	Induces miR‐181c‐5p to enhance osteogenesis by upregulating RUNX2 and mineralization via *Notch2* downregulation
Da W, 2020 [96]	Female SPF C57BL6/J mouse	60 mg/kg/day IP	60 mg/kg/day	Increases bone mass and citrate in OVX mice
	Mouse BMSC	1 and 10 µM	1 and 10 µM	Enhances mineralization and citrate release via ZIP‐1 upregulation
Zheng M, 2020 [97]	Human PDLSC	1 pM to 1 µM	1 pM to 10 nM	Suppresses osteogenesis via reduction of OPN, OCN, and mitochondrial functions
Xiao L, 2020 [98]	Mouse MC3T3‐E1 cell	10 µM	10 µM	GelMA‐DOPA@MT promotes osteogenesis and inhibits osteoblast apoptosis via SIRT3/SOD2
	Female SD OVX rat	50 mg/kg direct injection	50 mg/kg	GelMA‐DOPA@MT prevents OVX‐induced bone loss and promotes osteogenesis around the implant
Huang J, 2021 [99]	Female C57/BL OVX mouse	50 mg/kg/day IP	50 mg/kg/day	Increases bone volume fraction, COL1, and BMP‐2, combined with PEMF
Wu X, 2021 [100]	Male Wistar rat	534 and 1068 µg (1.67 and 3.34 mg/kg/day) oral	1.67 and 3.34 mg/kg/day	Reduces bone resorption and proinflammatory cytokine levels by decreasing TLR4, NF‐κB to TNF, IL‐1β, IL‐6, and RANKL, while increasing OCN
	Rat BMM	1 μM	1 µM	Enhances osteoblastogenesis and function
Wang X, 2021 [101]	hBMSC	100 µM	100 µM	Enhances osteogenesis by inhibiting circ_0003865 to repress *GAS1* via miR‐3653‐3p activation and suppressing osteoporosis
Han H, 2021 [102]	Female SD rat BMSC	100 µM	100 µM	Activates osteogenesis and abates adipogenesis via lncRNA H19/miR‐541‐3p/APN and Wnt/β‐catenin
Oliveira EA, 2022 [103]	Mouse MC3T3‐E1 cell	1 mM	1 mM	Increases osteoblast proliferation and viability but shows no effect when combined with Bio‐Gideä resorbable membranes
Zheng S, 2022 [104]	Rat BMSC	10 nM to 10 µM	100 nM	Enhances osteogenesis, angiogenesis (100 nM, > 1 µM, and 10 nM), and osteogenesis–angiogenesis coupling
	SD rat	10 and 50 mg/kg/day IP	10 and 50 mg/kg/day	Promotes osteoporotic bone repair in OVX rats
Xie Y, 2022 [105]	Human BMSC C57BL/6 mouse BMSC	1 µM	1 µM	Promotes osteoblastogenesis of senescent BMSC via NSD 2‐mediated chromatin remodeling
	C57 mouse	10 mg/kg SC twice a week	10 mg/kg	Ameliorates osteoporosis in aged mice via upregulation of NSD2
Chan YH, 2022 [114]	Rabbit dental pulp MSC	1, 10, and 100 µM	100 µM	Promotes osteogenesis via p38/ERK
	Rabbit	100 µM in HA/TCP implant	100 µM	HA/TCP enhances bone regeneration in calvarial defects
Guan H, 2022 [106]	Female SD rat	100 mg/kg/day oral	100 mg/kg/day	Increases bone mass in normal, perimenopausal, and postmenopausal osteoporotic rats by promoting osteogenesis
	C57BL/6J mouse BMSC	100 nM to 10 mM	100 µM	Increased *Col1a1*, *Runx2*, *Alpl*, *Alpl*, and *Bglap*
Li M, 2022 [107]	SD rat BMSC	5–200 µM	10, 50, and 100 µM	Suppresses ferroptosis via PI3K/AKT/mTOR
	SD rat SIOP model	50 mg/kg/day IP	50 mg/kg/day	Meliorates ferroptosis and osteoporosis
Munmun F, 2022 [44]	hMSC Mouse MSC	50 nM	50 nM	MEK1 and MEK5 in hMSCs and MTs, MEK1, and MEK5 in mouse MSCs are critical for melatonin‐induced osteoblastogenesis Protects against mouse MSC and osteoblast loss under oxidative stress
	Balb(c) female mouse	0.166 mg/kg/day IP	0.166 mg/kg/day	MEK1/2 and MEK5 regulate melatonin's effects on bone microarchitecture, biomechanics, and osteogenic proteins MEK1 and MEK5 influence melatonin's bone formation and rate
	Balb(c) (male and female) mouse	15 mg/L/day oral	15 mg/L/day	MEK1 and MEK5 mediate melatonin's effects on bone formation, decreasing rates in males and showing an increasing trend in females
Li TL, 2023 [108]	Female SD rat	30 mg/kg/day IP	30 mg/kg/day	Offers better bone mass protection with daytime administration
	Mouse MC3T3‐E1 cell	100 and 400 μM	100 μM	Enhances cell viability and reduces ROS production
Hu Y, 2023 [109]	Male SD rat BMSC	50 and 100 μM	100 μM	Suppresses inflammatory response via NF‐κB Counteracts TNF‐α‐induced suppression of osteogenesis and inflammation
Huang X, 2023 [47]	OVX SD rat BMSC	1 and 100 μM	100 μM	Activates SIRT1 to enhance osteogenesis and mineralization by upregulating RUNX2 and suppress adipogenesis by downregulating PPARγ
	Female OVX SD rat	10 mg/kg/day IV	10 mg/kg/day	Reverses the switch from osteogenesis to adipogenesis via SIRT1 activation

Abbreviations: ALP: alkaline phosphatase; AMPK: AMSC: adipose‐derived mesenchymal stem cell; BM: bone marrow; BMD: bone mineral density; BMM: bone marrow derived macrophage (monocyte); BMP: bone morphogenic protein; BMSC: bone marrow mesenchymal stem cell; C2C12 cell: mouse pre‐myoblast cell; COL1: collagen type I; CRE: cAMP‐responsive element; CTX: C‐terminal telopeptide; DM: diabetes mellitus; EGFR: epidermal growth factor receptor; ER: estrogen receptor; ERK: extracellular signal‐regulated kinase; FOXO3a: forkhead box O3a; GLUT4: glucose transporter type 4; HA/TCP: hydroxyapatite/tricalcium phosphate; hFOB 1.19: human fetal osteoblastic cell; hOB cell: human jawbone‐derived osteoblastic cell; HOB‐M cell: human bone cell; hPDLC: human periodontal ligament cells; IGF: insulin‐like growth factor; IκB: a specific inhibitor of the NF‐κB transcription factor; IKK: IκB kinase; nuclear factor of activated T‐cells, cytoplasmic 1; IL: interleukin; IP: intraperitoneal injection; IV: intravenous injection; JNK: c‐Jun N‐terminal kinase; LPS: lipopolysaccharide; MAPK: mitogen‐activated protein kinase; MC3T3‐E1: mouse preosteoblast cell; MEK: MAPK/ERK kinase; MMTV/*Neu*: unactivated rat *Neu* (*c‐ErbB2*) transgene [FVB/N‐Tg(MMTVneu)202 Mul/J] with wild‐type FVB/N females; MMTV‐HER2/*neu* mouse: mouse mammary tumor virus‐wild‐type human Her‐2, c‐ErbB‐2 transgenic mouse; MSC: mesenchymal stem (stromal) cell; MSDK, In Vitro: 50 nM melatonin, 191.5 µM strontium, 26 nM Vit. D3, and 18.5 nM Vit. K2; MSDK, In Vivo: 5 mg melatonin, 450 mg strontium, 2000 IU Vit. D3, and 60 µg Vit. K2; MSK1: mitogen and stress‐activated protein kinase; MT: melatonin receptor; mTOR: mammalian target rapamycin; NF‐κB: nuclear factor κ‐light‐chain‐enhancer of activated B cells; NLRP: nucleotide‐binding domain leucine‐rich repeat (NLR) and pyrin domain‐containing receptor; Notch2: neurogenic locus notch homolog protein 2; NPY: neuropeptide Y; NPY1R: NPY receptor; NSD2: nuclear receptor binding SET domain protein 2; OCN: osteocalcin; OPG: osteoprotegerin; OVX: ovariectomized; p‐: phosphorylated; p90RSK: 90‐kDa ribosomal S6 kinase; PBM: peripheral blood monocyte (macrophage); PDL(S)C: periodontal ligament (stem) cell; PEMF: pulsed electromagnetic field; PIN1: protein never in mitosis gene A interacting‐1; PI3K: phosphatidylinositol 3‐kinase; PKA: protein kinase A; PLGA: poly (lactic‐co‐glycolic acid) microspheres; PPARγ: peroxisome proliferator‐activated receptor γ; Prkd1: protein kinase D1; PRMT: protein arginine methyltransferase; RANKL: receptor activator of nuclear factor‐κB ligand; RCT: randomized controlled trial; ROS: reactive oxygen species; ROS17/2.8 cell: rat osteoblast‐like cell; NX: runt‐related transcription factor; SC: subcutaneous injection; SD rat: Sprague‐Dawley rat; SIOP: steroid‐induced osteoporosis; SIRT1: silent information regulator type 1; SMAD: suppressor of mothers against decapentaplegic; SMURF1: SMAD‐specific E3 ubiquitin protein ligase 1; STIM1: stromal‐interacting molecule 1; SV‐HFO cell: human osteoblastic cell; Ti: titanium; TNF: tumor necrosis factor; TRACP: Wnt: tartrate‐resistant acid phosphatase; wingless‐related integration site; TRAF6: tumor necrosis factor receptor‐associated factor 6; vBMD: volumetric bone mineral density; ZIP‐1 zinc transporter.

#### 4.1.1 In Mouse MC3T3‐E1 Preosteoblast Cells

##### 4.1.1.1 Physiological Concentrations (nM)

In MC3T3‐E1 cells, melatonin (1–100 nM) upregulated essential bone formation genes, including bone sialoprotein (*Bsp*), osteocalcin [*Ocn*, also known as osteoblast‐specific bone gamma‐carboxyglutamic acid (Gla) protein, *Bglap*], osteopontin (*Opn*), and *Alp* [35]. It promoted osteoblastic differentiation, proliferation, viability, and mineralization. At 50 nM, it enhanced these effects by activating the BMP/ERK/wingless‐related integration site (Wnt) signaling pathway [35, 69].

##### 4.1.1.2 Pharmacological Concentrations (µM–mM)

Melatonin at pharmacological concentrations exerts protective and therapeutic effects on bone health through multiple pathways. At 1 µM, it counteracted glucocorticoid‐induced suppression of osteoblast differentiation by activating the PI3K/AKT and BMP/SMAD signaling [110]. At 1 and 10 µM, it enhanced mineralization and citrate release via zinc transporter (ZIP‐1) upregulation [96]. A GelMA‐DOPA@MT hydrogel system delivering 10 µM melatonin promoted osteogenesis and protected preosteoblasts from oxidative stress‐induced apoptosis via the SIRT3/superoxide dismutase (SOD)2 pathway, reducing bone loss in ovariectomized (OVX) rats and minimizing osteoblast apoptosis around implants, thereby potentially lowering implant loosening risk in osteoporotic patients [98].

At 100 µM, melatonin prevented ferroptosis in osteoblasts, a key factor in type 2 diabetic osteoporosis, by activating the nuclear factor erythroid 2‐related factor 2 (Nrf2)/heme oxygenase‐1 (HO‐1) pathway In Vitro [92]. Intraperitoneal dose of 10 or 50 mg/kg improved bone structure and mass In Vivo, while promoting osteoblast differentiation under hypoxia through p38 and protein kinase D1 (Prkd1) activation [111]. At 5–500 µM, it suppressed osteoclastogenesis by downregulating RANKL and OPG expression in preosteoblasts [20]. Overall, melatonin enhanced osteoblast function and signaling in a concentration‐dependent manner.

At 1 mM, melatonin enhanced osteoblast proliferation and viability but showed no added effect with Bio‐Gideä resorbable membranes [103]. It improved osteoblast anabolism via p‐ERK and MnSOD under fluid shear stress and enhanced ERK/Akt/mammalian target rapamycin (mTOR) signaling in cilia‐less MC3T3‐E1 cells [58, 80]. Melatonin also activated both canonical and noncanonical Wnt4 pathways, promoting bone formation and counteracting tumor necrosis factor‐α (TNF‐α)/NF‐κB induced inhibition [59]. However, at higher concentrations (2.5 mM), melatonin exhibited inhibitory effects, reducing metabolic activity and RNA content and increasing cytotoxicity [74]. At 4 mM, it induced mitochondrial apoptosis in osteoblasts through stromal‐interacting molecule 1 (STIM1) and ERK‐mediated calcium elevation [91].

#### 4.1.2 In Murine MSCs

##### 4.1.2.1 Physiological Concentrations (nM)

Melatonin (20–200 pg/mL) promoted osteogenesis in rat BMSCs but not in adipose‐derived MSCs (AMSCs) [66]. In Sprague‐Dawley (SD) rat BMSCs, it enhanced cell proliferation, osteogenesis, and angiogenesis non‐dose‐dependently, with 100 nM being optimal, followed by 1 μM and 10 nM [112]. Additionally, melatonin at 100 nM improved osteogenesis–angiogenesis coupling in BMSCs, supporting bone repair in OVX rats.

At 50 nM, melatonin promoted osteoblastogenesis in human and mouse MSCs via MEK1/2 and MEK5 pathways, which also protected against oxidative stress and regulated bone microarchitecture, biomechanics, and protein expression [44]. MEK1 and MEK5 had distinct roles in bone formation. In mice, a 3‐month oral melatonin (15 mg/L/day) revealed sex‐specific effects, reducing bone formation rate in males and trending upward in females.

##### 4.1.2.2 Pharmacological Concentrations (µM–mM)

At pharmacological concentrations, melatonin promoted osteogenesis and safeguarded bone health through multiple mechanisms. At 1 µM, it enhanced BMP‐4‐induced osteogenesis in mouse pre‐myoblast C2C12 cells through SMAD‐dependent osterix upregulation and protein kinase A (PKA) and PKC‐regulated stabilization [51]. In mouse BMSCs, it facilitated osteoblastogenesis in senescent BMSCs through NSD 2‐mediated chromatin remodeling [23]. In cocultures of BMSCs and AMSCs, 5 µM melatonin increased B‐cell lymphoma 2 (BCL2) expression, reduced BCL2‐associated X (BAX) expression and apoptosis, and enhanced cell survival and osteogenesis by upregulating OCN levels [87]. In mouse BMSCs, at 10 µM, it upregulated microRNA (miRNA/miR)‐92b‐5p to suppress intracellular adhesion molecule‐1 (ICAM‐1), thereby facilitating osteogenesis [90]. In a rat steroid‐induced osteoporosis (SIOP) model, intraperitoneal melatonin (50 mg/kg), combined with treating rat BMSCs with 10–100 µM melatonin, prevented ferroptosis, a form of cell death induced by iron and ROS, through PI3K/AKT/mTOR activation, reducing steroid‐induced bone loss [107].

At 100 µM, melatonin attenuated inflammatory responses in SD rat BMSCs by inhibiting NF‐κB and restoring osteogenesis and TNF‐α‐induced inflammation [109]. In OVX C57BL/6J mouse BMSCs, it downregulated nucleotide‐binding domain and the leucine‐rich repeat pyrin 3 domain (NLRP3) inflammasome and interleukin (IL)‐1β expression, effects partially reversed by Dickkopf‐1 (DKK1) [113]. Additionally, it enhanced osteogenesis while suppressing adipogenesis in SD rat BMSCs through the lncRNA H19/miR‐541‐3p/APN axis and Wnt/β‐catenin activation [102]. At 2 mM, melatonin promoted neuropeptide Y (NPY) and its receptor NPY1R expression, facilitating MSC proliferation, migration, and osteoblastic differentiation in male SD rats, effects blocked by the NPY1R antagonist BIBP3226 [83].

#### 4.1.3 In Rat Osteoblast‐Like ROS17/2.8 Cells and Rabbit Dental Pulp‐Derived MSCs

In ROS17/2.8 cells, melatonin at 50 nM enhanced osteoblast differentiation and mineralization [35], while 100 nM reduced fatty acid‐induced triglyceride accumulation [65]. At 100 µM, when integrated into hydroxyapatite/tricalcium phosphate (HA/TCP) ceramic powder, melatonin promoted cell proliferation, osteogenesis, and osteogenic gene expression in rabbit dental pulp‐derived MSCs through p38/ERK MAPK activation, enhancing bone regeneration in rabbit calvarial bone defects [114].

#### 4.1.4 In Human MSCs and Osteoblastic Cells

##### 4.1.4.1 Physiological Concentrations (nM)

At 50 nM, melatonin enhanced ALP activity in differentiating human MSCs cultured in osteogenic medium through the MT_2_R/Gi/β‐arrestin/MEK/ERK1/2 pathway [36]. It promoted osteoblast differentiation and bone formation via MT receptors, involving epidermal growth factor receptors (EGFRs), metalloproteinases, and clathrin‐mediated endocytosis, independently of PKA [42]. It also facilitated chondrogenesis by upregulating miR‐526b‐3p and miR‐590‐5p, enhancing SMAD1 phosphorylation via *Smad7* [52, 85]. In cocultures of human MSCs (hMSCs) and peripheral blood monocytes (PBMs), the combination of melatonin (50 nM), strontium citrate, vitamin D3, and vitamin K2 enhanced osteoblastogenesis, increased OPG levels, reduced osteoclastogenesis and RANKL expression, and modulated ERK1/2, ERK5, runt‐related transcription factor 2 (RUNX2), peroxisome proliferator‐activated receptor γ (PPARγ), and glucose transporter type 4 (GLUT4) [37]. Similarly, in human MSCs and peripheral blood mononuclear cells (PBMCs) cocultures, melatonin at 50 nM increased the OPG/RANKL ratio via MT_2_, MEK/ERK1/2, and MEK/ERK5 pathways [7]. In transwell osteoblast models, it upregulated RUNX2 and NF‐κB while downregulating PPARγ, GLUT4, insulin receptor β, and integrin β1, promoting the differentiation of AMSCs into osteoblasts similarly to BMSCs. MEK1/2 and MEK5 were critical for melatonin's osteoblastogenesis in human and mouse MSCs, and they protected against oxidative stress‐induced cell and osteoblast loss [44].

##### 4.1.4.2 Pharmacological Concentrations (µM–mM)

At 1 μM and 100 μM, melatonin promoted osteogenesis of hMSC in an IL‐1β‐induced inflammatory environment by increasing proliferation and cell viability while reducing ROS via SOD upregulation [115]. At 10 μM and 100 μM, it mitigated oxidative stress by activating AMP‐activated protein kinase (AMPK) and enhancing forkhead box O3a (FOXO3a) and RUNX2 [116].

At 100 µM, melatonin stimulated human osteoblastic differentiation while inhibiting adipogenesis by repressing PPARγ and activating RUNX2 [67]. It protected hMSCs from oxidate stress H_2_O_2_‐induced premature senescence by downregulating p16^INK4a^ and upregulating SIRT1 via MT, thereby restoring osteogenic capacity [76]. Melatonin protected SMAD1 from TNF‐α‐induced degradation by restoring SMAD‐specific E3 ubiquitin protein ligase 1 (SMURF1), thus preserving BMP–SMAD1 signaling and supporting osteogenesis in hMSCs [117]. It also reversed TNF‐α‐induced ROS generation and osteogenesis inhibition, downregulating the NF‐κB pathway by inhibiting p65 phosphorylation and blocking IκB‐α degradation [89]. Furthermore, melatonin enhanced osteogenesis of hBMSCs by sponging circ_0003865, repressing miR‐3653‐3p, and leading to *Gas1* gene activation and osteoporosis prevention in a mouse model [101].

At 1 mM, melatonin promoted bone formation through both canonical Wnt4‐Fzd1/6‐LRP5/6‐β‐catenin and noncanonical Wnt4‐Fzd2‐JNKp38 pathways, counteracting TNF‐α/NF‐κB‐mediated osteogenesis suppression [59]. At 1 µM, it promoted osteoblastogenesis in senescent BMSCs through NSD2‐mediated chromatin remodeling [23]. In contrast, at 1 pM to 1 µM, melatonin inhibited osteogenesis in human periodontal ligament stem cells by downregulating OPN and OCN [118], emphasizing its dose‐ and cell type‐specific molecular mechanisms through which melatonin influences bone health [1, 14].

At 100 µM, melatonin prevented ethanol‐induced senescence and osteoclastic differentiation in human cementoblasts and periodontal ligament cells via mTOR, AMPK, MAPK, and NFATc‐1 pathways, with effects inhibited by blocking the protein never in mitosis gene A interacting‐1 (PIN1) [82]. A calcium aluminate‐melatonin scaffold with 1 mg/mL of melatonin enhanced cell adhesion, viability, and proliferation in human osteoblasts, promoted hMSCs differentiation into osteoblasts, and boosted osteoconductive and osteoinductive properties without affecting NIH3T3 fibroblasts [70]. Poly(lactic‐co‐glycolic acid) (PLGA) microspheres loaded with 10 mg melatonin provided sustained release, enhancing ALP activity and osteoblast differentiation markers such as RUNX2, OPN, and OCN [73]. In OVX rats, melatonin at 25 µg/mL/day amplified estradiol's protective effects [119], underscoring its multifaceted role in supporting bone health, especially in combination with hormones such as PTH, estrogen, and calcitonin.

#### 4.1.5 In Human Osteoblastic hFOB 1.19 Cells

At 10 nM to 100 µM, melatonin stimulated osteoblast proliferation via the cascade involving c‐Raf, MEK1/2, ERK1/2, the 90‐kDa ribosomal S6 kinase (p90RSK), and mitogen‐ and stress‐activated protein kinase 1 (MSK1) [75]. Conversely, at 1 mM, it inhibited the growth of hFOB 1.19 cells by causing cell cycle arrest in the G1 and G2/M phases [68]. At 1 nM and 10 µM, it slightly enhanced cell cycle progression, especially in the G2/M phase. At 1 µM, melatonin‐induced miR‐181c‐5p promoted osteogenesis by upregulating RUNX2 expression through TGF‐β1‐induced ERK1/2 activation and calcification via neurogenic locus notch homolog protein 2 (*Notch2*) downregulation in human jawbone‐derived osteoblastic (hOB) cells [95]. Melatonin (5–100 µM) stimulated osteoblast and bone cell proliferation and COL1 synthesis without influencing OCN production or ALP activity, while at 10 µM and 1 mM, it inhibited autophagy [62, 120]. At 2 mM, melatonin modulated cAMP‐responsive element (CRE)‐dependent gene transcription to support proliferation by concurrently activating Src and PKA [84], while at 4 mM, it induced apoptosis in osteoblasts through miR‐590‐3p targeting septin 7 [86].

#### 4.1.6 In Murine

In mice, intraperitoneal administration of melatonin (5 or 50 mg/kg/day) increased BMD by 36%, bone mass by 49%, and trabecular thickness by 19% [20], while reducing osteoclast surface by 74% and number by 76%, primarily by downregulating the RANKL/OPL ratio. In OVX rats, melatonin administered at 10 mg/kg/day subcutaneously mitigated the increase in inducible NO synthase (iNOS) expression, apoptosis in the nucleus pulposus and epiphyseal cartilage, and osteoclasts while preserving osteoblasts [63], At 10 and 30 mg/kg/day, it reversed reductions in trabecular thickness and area in the vertebrae and femurs, along with cortical thickness in the femurs but did not affect osteoblast or osteoclast numbers, trabecular numbers, or hydroxyproline levels [64].

Intraperitoneal melatonin (100 mg/kg) promoted cortical bone formation, enhanced bone mass via MT_2_ activation, reversed OVX‐induced bone loss, and restored overall bone quality [43]. Long‐term oral melatonin treatment (0.05 mg/night for 1 year) increased bone density by 20% and upregulated osteoblast‐related genes [71]. Oral melatonin (15 mg/mL/day) elevated the OPG/RANKL (25 kDa) ratio and p‐ERK1/2, p‐ERK5, and RUNX2 levels in bone, mitigating OVX‐induced osteoporosis [7]. Dietary oral melatonin (15 mg/mL or 10 mg/kg) improved bone parameters in aged rats [7, 34]. Intraperitoneal melatonin (10 and 50 mg/kg) enhanced osteogenesis and elevated serum ALP and OCN levels in OVX mice, by inhibiting the NLRP3 inflammasome via Wnt/β‐catenin signaling [113].

Melatonin (50 mg/kg/day, intraperitoneally) alleviated mitochondrial oxidative stress in OVX rats and MC3T3‐E1 cells by modulating NADPH oxidase 2 and cytochrome C levels via the SIRT3/SOD2 pathway, enhancing osteogenesis [88]. In OVX mice, melatonin (50 and 60 mg/kg/day, intraperitoneally) altered the cytokine framework, increased bone mass, and elevated citrate content [65]. Combined with low‐frequency pulsed electromagnetic field therapy, intraperitoneal melatonin at 50 mg/kg/day boosted bone volume fraction, enhanced COL1 and BMP‐2 expression, reduced the bone surface‐to‐volume ratio, and decreased osteoclast numbers in the metaphysis of female C57BL/6 mice [99]. Subcutaneous melatonin (10 mg/kg, twice weekly) alleviated osteoporosis in aged mice by upregulating nuclear receptor‐binding SET domain protein 2 (NSD2) [23]. Furthermore, intraperitoneal melatonin (50 mg/kg/day) promoted osteoblast differentiation in aged rat BMSCs [79].

Intraperitoneal melatonin (5 or 50 mg/kg/day) counteracted titanium (Ti)‐induced osteolysis and stimulated bone formation via the Wnt/β‐catenin pathway in male C57BL/6 J mouse calvarial models and mouse MSCs [78]. In type 2 diabetic SD rats, melatonin delayed diabetes‐induced osteoporosis and reduced autophagy levels, with 50 mg/kg/day showing greater efficacy than 100 mg/kg/day by inhibiting ERK [120]. In type 1 diabetic SD rats, melatonin enhanced short‐term bone healing, antioxidant defenses, and bone mass and quality by reducing bone resorption and proinflammatory cytokines [77]. Daily oral melatonin (5 mg/kg) supported the recovery of impaired bone repair due to pinealectomy during osseointegration [81]. It also increased OCN levels both In Vivo (1.67 and 3.34 mg/kg/day) and in vitro (1 µM) [100]. In an SD rat SIOP model, intraperitoneal melatonin (50 mg/kg/day) mitigated ferroptosis and osteoporosis [107].

In OVX rats and BMSCs, oral melatonin at 100 mg/kg/day or at 100 µM In Vitro prevented bone loss and promoted osteogenesis by upregulating *Col1a1*, *Runx2*, *Alpl*, and *Bglap* [106]. Intravenous melatonin (10 mg/kg/day) preserved bone mass and architecture and shifts differentiation toward osteogenesis via *Runx2* upregulation, PPARγ downregulation, and SIRT1 activation [47]. Intraperitoneal melatonin (30 mg/kg/day) was more effective during daytime administration in increasing bone mass in OVX rats. In MC3T3‐E1 cells, low‐dose melatonin (100 µM) enhanced cell viability and suppressed ROS compared with a high dose (400 µM), highlighting the importance of timing and dosage in osteoporosis treatment [108].

#### 4.1.7 In Humans

In postmenopausal women with osteopenia, a combination of melatonin (5 mg), strontium citrate, vitamin D3, and vitamin K2 improved BMD, bone turnover markers (BTMs), mood, and sleep quality [37]. A 6‐month trial of 3 mg melatonin nightly in women aged 45–54 normalized BTMs, improved physical health, and extended cycle intervals [72]. A 1‐year randomized controlled trial (RCT) in women aged 56–73 showed that nightly melatonin increased femoral neck BMD dose‐dependently (0.5% at 1 mg; 2.3% at 3 mg) [38]. At 3 mg/day, melatonin also improved tibial trabecular thickness (2.2%), spinal volumetric BMD (3.6%), and reduced 24‐h urinary calcium excretion (12.2%), with no significant effects on other sites or BTMs.

### 4.2 Effects of Melatonin on Osteoclasts

#### 4.2.1 In Murine and Rats

##### 4.2.1.1 Physiological Concentrations (nM) In Vitro and In Vivo Studies

Melatonin (10 and 100 nM) inhibited osteoclast formation in mouse BMSCs by modulating RANKL in indirect‐contact cocultured with osteoclast precursors but not in the direct‐contact cocultured system [45] (Table 2). In osteoporotic C57/BL/6J mice, oral melatonin (10 and 100 mg/kg per day) enhanced bone mass by counteracting OVX‐induced changes in brain and muscle ARNT‐like 1 (*Bmal1*) and osteoclast markers. In hMSC and pre‐osteoclast transwell cocultures, melatonin (50 nM) regulated osteoclast differentiation via MEK1 and MEK5 pathways, reducing osteoclast numbers in female mice while altering osteoclast‐related protein expression through MEK1/2 and MEK5 [44].

**Table 2:** Effects of melatonin on osteoclastogenesis.

Reference	Model	Testing dose and route	Effective dose	Evidence and mechanism
Ladizesky MG, 2001 [121]	Female Wistar rat	500 µg/day oral	500 µg/day	Modifies bone remodeling with adequate estradiol in OVX rats
Ostrowska Z, 2002 [122]	Female rat	50 µg/100 g of body mass IP	50 µg/100 g of body mass	Suppresses OVX‐induced bone marker changes but only partially offsets pinealectomy‐induced bone metabolism changes
Koyama H, 2002 [20]	Male *ddy* mouse	1, 5, and 50 mg/kg/day IP	5 and 50 mg/kg/day	Increases BMD by 36%, bone volume per tissue volume by 49%, and trabecular thickness by 19% via inhibition of bone resorption
	Mouse BM cell Rabbit osteoclast	1–500 µM	5–500 µM	Reduces resorption pit number and area in osteoclasts from mouse BM cells, but not rabbit osteoclasts
Suzuki N, 2002 [61]	Female matured goldfish	1 nM to 10 µM	10 nM to 10 µM	Affects scale osteoclastic cells by reducing TRACP via reduction of ER and IGF‐1 mRNAs
Ladizesky MG, 2003 [119]	Female Wistar rat	25 µg/mL/day oral	25 µg/mL/day	Reverses bone remodeling disruption and enhances estradiol's protective effects on bone in OVX rats
Oktem G, 2006 [63]	Female Wistar rat	10 mg/kg/day SC	10 mg/kg/day	Rescues the increase of iNOS and apoptotic cell numbers in the nucleus pulposus and epiphyseal cartilage and the increase of osteoclasts
Histing T, 2012 [123]	CD‐1 mouse	50 mg/kg/day IP	50 mg/kg/day	Decreases RANKL, COL1, and the number of TRAP‐positive osteoclasts to inhibit bone resorption
Satué M, 2015 [74]	Mouse RAW 264.7 cell	0.1 mM	0.1 mM	Decreases *Mmp9* gene expression while promoting ERK1/2
Ping Z, 2017 [124]	Male C57BL/6 mouse calvarial model	5 and 50 mg/kg/day IP	5 and 50 mg/kg/day	Suppresses wear debris‐induced bone resorption and inflammatory cytokine expressions
	Mouse C57BL/6 BMM	0.1–2 mM	0.5 and 1.0 mM	Suppresses RANKL‐induced osteoclast differentiation, F‐actin ring formation, and osteoclastic resorption
	Mouse RAW 264.7 cell	1.0 mM	1.0 mM	Inhibits RANKL‐induced osteoclastic formation, NF‐κB/NFATc1, and c‐fos via reduction of p‐IκB‐α and p‐p65 but not on p‐IKKα, MAPK, or PI3K/AKT activation
Zhou L, 2017 [19]	Mouse C57BL/6 BMM	0.01–10 nM to 1–100 μM	1–100 μM	Suppress BMM osteoclastogenesis via the ROS‐mediated but not SIRT1‐independent pathway
Ping Z, 2017 [78]	Male C57BL/6J mouse calvarial model	5 and 50 mg/kg/day IP	5 and 50 mg/kg/day	Suppresses Ti‐induced osteolysis by modulating RANKL/OPG via Wnt/β‐catenin
	Mouse MSC	1–100 µM	100 µM	Modulates RANKL/OPG by activating Wnt/β‐catenin
Maria S, 2017 [37]	Postmenopausal osteopenic woman	5 mg (MSDK, In Vivo)	5 mg	MSDK decreases CTX/P1NP MSDK improves mood and sleep quality
	Human MSC and PBM	50 nM (MSDK, In Vitro)	50 nM	MSDK decreases in osteoclastogenesis and RANKL MSDK suppresses adipogenesis by inhibiting PPARγ and GLUT4
Kim HJ, 2017 [125]	Mouse BMM	100–800 µM	200 and 500 nM	Decreases RANKL‐induced osteoclastogenesis by inhibiting NF‐κB and NFATc1, but not affecting ERK, JNK, and p38
Maria S, 2018 [7]	Human MSC and PBM	50 nM	50 nM	Increases OPG/RANKL by inhibiting RANKL via MT_2_, MEK/ERK1/2, and MEK/ERK5
	Female MMTV‐HER2/*neu* mouse	15 mg/mL/day oral	15 mg/mL/day	Increase in the OPG/RANKL (25 kDa) ratio, p‐ERK1/2, p‐ERK5, and RUNX2 in mouse bone
Kobayashi‐Sun J, 2020 [93]	Zebrafish scale	100 nM	100 nM	Suppresses osteoblast and osteoclast differentiation via inhibition of epidermal ERK
Zhou Y, 2020 [45]	Female C57/BL mouse	10 and 100 mg/kg/day oral	10 and 100 mg/kg/day	Transforms the cytokine framework and improves bone mass in OVX mice
	Mouse BMSC + BMM	10 and 100 nM	10 and 100 nM	Downregulates osteoclastogenesis via RANKL paracrine secretion, not in a cell‐to‐cell direct contact manner
Huang J, 2021 [99]	Female C57/BL mouse	50 mg/kg/day IP	50 mg/kg/day	Decreases the bone surface/bone volume ratio and osteoclast numbers in the metaphysis, combined with pulsed electromagnetic field in OVX mice
Jarrar H, 2021 [126]	Mouse RAW 264.7 cell	800 µM	800 µM	Attenuates osteoclast differentiation induced by BMP‐2
Liu PI, 2021 [127]	Mouse RAW 264.7 cell	0.1–1 mM	0.7 mM	Decreases osteoclast differentiation and functioning, enhances osteoclast apoptosis, and inhibits cancer‐secreted RANKL by reducing p38
Wu X, 2021 [100]	Male Wistar rat	1.67 and 3.34 mg/kg/day oral	1.67 and 3.34 mg/kg/day	Reduces bone resorption, osteoclast numbers, and proinflammatory cytokines by decreasing TLR4 and NF‐κB to TNF, IL‐1β, IL‐6, and RANKL
	Rat PBM	1 μM	1 µM	Suppresses osteoclast formation and function Suppresses LPS‐induced TNF, IL‐1β, and IL‐6 by inhibiting TLR4/NF‐κB
Choi JH, 2021 [128]	Mouse BMM	500 μM	500 μM	Suppresses osteoclast differentiation by inhibiting RANKL‐induced TRAF6/JNK and PRMT1, and NF‐κB via p65 interference The signaling cascades are MT‐independent
	Female C57BL/6 mouse	5 and 25 mg/kg/2 days IP	5 and 25 mg/kg/2 days	Suppresses PRMT1 in OVX mice bones to attenuate osteoclast‐mediated bone resorption
Kim SS, 2022 [129]	Mouse RAW264.7 cell	10–2000 µM	300 µM	Ameliorates RANKL‐induced osteoclast differentiation and fusion by attenuating Atp6v0d2 and DC‐STAMP via MAPK and NFATc1
Munmun F, 2022 [44]	hMSC + preosteoclast	50 nM	50 nM	MEK1 and MEK5 for melatonin‐induced osteoclastogenesis in transwell cocultures
	Balb(c) female mouse	0.166 mg/kg/day IP	0.166 mg/kg/day	MEK1/2 and MEK5 involve melatonin's osteoclast‐regulating proteins MEK1 and MEK5 are involved in melatonin's decrease in osteoclast number
Wang X, 2023 [130]	Mouse RAW264.7 cell	0.01–0.5 mM	0.3 mM	Enhances RAW264.7 apoptosis via BMAL1/ROS/MAPK‐p38
	Female C57BL/6 mouse	30 mg/kg/day IG	30 mg/kg/day	Improves bone loss in OVX mice

Abbreviations: A549: lung cancer cell line; ALP: alkaline phosphatase; AMSC: adipose‐derived mesenchymal stem cell; BM: bone marrow; BMAL: brain and muscle ARNT‐like; BMM: bone marrow derived macrophage (monocyte); ER: alkaline phosphatase; ERK: extracellular signal‐regulated kinase; GLUT4: glucose transporter type 4; IG: intragastrical injection; IGF: insulin‐like growth factor; IκB: a specific inhibitor of the NF‐κB transcription factor; IKK: IκB kinase; nuclear factor of activated T‐cells, cytoplasmic 1; IP: intraperitoneal injection; IV: intravenous injection; JNK: c‐Jun N‐terminal kinase; LPS: lipopolysaccharide; MMTV‐HER2/*neu*: mouse mammary tumor virus‐wild‐type human Her‐2, c‐ErbB‐2; MT: melatonin receptor; MSC: mesenchymal stem (stromal) cell; MSDK, In Vitro: 50 nM melatonin, 191.5 µM strontium, 26 nM Vit. D3, and 18.5 nM Vit. K2; MSDK, In Vivo: 5 mg melatonin, 450 mg strontium, 2000 IU Vit. D3, and 60 µg Vit. K2; NF‐κB: nuclear factor κ‐light‐chain‐enhancer of activated B cells; OPG: osteoprotegerin; OVX: ovariectomized; p‐: phosphorylated; PBM: peripheral blood monocyte (macrophage); PC‐3: osteolytic prostate cancer cell line; PEMF: pulsed electromagnetic field; PPARγ: peroxisome proliferator‐activated receptor γ; PRMT: protein arginine methyltransferase; RANKL: receptor activator of nuclear factor‐κB ligand; RAW264.7: mouse mononuclear macrophage leukemia cell; ROS: reactive oxygen species; RUNX: runt‐related transcription factor; SC: subcutaneous injection; SIRT1: silent information regulator type 1; TRAF6: tumor necrosis factor receptor‐associated factor 6; TRAP: tartrate‐resistant acid phosphatase; TRACP: tartrate‐resistant acid phosphatase.

##### 4.2.1.2 Pharmacological Concentrations (µM–mM) In Vitro and In Vivo Studies

Melatonin (1–100 µM) suppressed osteoclastogenesis in C57BL/6 mouse BMMs through a ROS‐mediated, SIRT1‐independent mechanism, while 0.1–10 nM had no effect [19]. It inhibited osteoclastogenesis by regulating the RANKL/OPG ratio through Wnt/β‐catenin activation, both In Vitro of 100 µM and In Vivo of 5 and 50 mg/kg/day intraperitoneally [78]. In mouse BMMs, melatonin (200 and 500 µM) suppressed RANKL‐induced osteoclast differentiation by inhibiting NF‐κB and NFATc1 without affecting MAPK pathways [125].

In mouse osteoclast precursor RAW 264.7 cells, melatonin (300 µM) reduced RANKL‐mediated osteoclast differentiation and maturation by downregulating Atp6v0d2 and DC‐STAMP through MAPK and NFATc1 inactivation pathways [129]. It also induced apoptosis by lowering ROS through *Bmal1* gene upregulation, suppressing ROS activity, and inhibiting p38 MAPK phosphorylation [130]. In mouse bone marrow‐derived osteoclasts, melatonin (5–500 µM) reduced the number and size of resorption pits by downregulating the RANKL/OPG ratio in osteoblastic cells, an effect absent in rabbit osteoclasts [20]. Intraperitoneal melatonin (5 and 50 mg/kg/day) improved bone health by increasing BMD (36%), bone volume per tissue volume (49%), and trabecular thickness (19%) via bone resorption inhibition in mice. Melatonin (500 µM) suppressed osteoclast differentiation in mouse BMMs by inhibiting RANKL‐induced activation of TNF receptor‐associated factor 6 (TRAF6), JNK, PRMT1, and NF‐κB signaling independently of MT receptors [128]. Additionally, melatonin (5 and 25 mg/kg every 2 days) reduced osteoclast‐mediated bone loss in OVX mice by inhibiting protein arginine methyltransferase 1 (PRMT1).

In RAW 264.7 cells, melatonin (700 µM) induced osteoclast apoptosis and suppressed cancer cell‐stimulated RANKL signaling through p38 inhibition [127], while at 800 µM, it attenuated BMP‐2‐induced osteoclastogenesis [126]. In male C57BL/6 mice (5 and 50 mg/kg/day, intraperitoneally) and BMMs (0.5 and 1.0 mM), melatonin reduced wear debris‐induced bone resorption and inflammatory cytokine production by inhibiting RANKL‐stimulated NF‐κB/NFATc1 and c‐Fos pathways [124]. Additionally, it (1.0 mM) decreased IκB‐α and p65 phosphorylation in RAW 264.7 cells without affecting IKKα, MAPK, or PI3K/AKT activation. As shown in a murine calvarial model, melatonin (5 and 50 mg/kg/day) mitigated bone loss and inflammatory responses.

In OVX rats with sufficient estradiol levels, oral melatonin (25 µg/mL/day or 500 µg/day) counteracted disruptions in bone remodeling and enhanced estradiol's protective effects on bone health [119, 121]. Intraperitoneal melatonin (50 µg/100 g) mitigated bone marker alterations caused by OVX but only partially reversed pinealectomy‐induced changes in bone metabolism [122]. In CD‐1 mice, intraperitoneal melatonin (50 mg/kg/day) reduced RANKL and COL1 expression and TRAP‐positive osteoclast numbers, indicating its ability to increase bone mass by inhibiting bone resorption rather than promoting bone formation [123]. Additionally, melatonin decreased osteoclastic numbers and activity to ameliorate bone resorption while diminishing LPS‐induced proinflammatory cytokines such as TNF, IL‐1β, and IL‐6 via inhibition of the TLR4/NF‐κB signaling pathway in male Wistar rats (1.67 and 3.34 mg/kg/day) and rat peripheral blood mononuclear cells (PBMs) (1 µM), mitigating inflammation and bone loss in rats [100].

#### 4.2.2 In Humans

The combination of melatonin (5 mg), strontium citrate, vitamin D3, and vitamin K2 (MSDK) enhanced bone density and BTMs in postmenopausal women with osteopenia by inhibiting osteoclast formation [37]. This effect is achieved by regulating RANKL and OPG synthesis and release from osteoblasts and promoting a healthier balance between bone resorption and formation.

## 5 Effects of Melatonin on Osteoporosis

Administering melatonin to normalize nocturnal levels helps protect bone health [40]. It supports bone metabolism by stimulating osteogenesis, inhibiting osteoclastogenesis, improving BMD, and potentially reducing the risk of fractures and osteoporosis [12, 38]. Melatonin generally has a mild side‐effect profile compared with hypnotics [131]; however, concerns about potential fracture risk from overdosing warrant further investigation, including its effects on postural stability, muscle strength, quality of life, and sleep [131, 132, 133]. Effective at up to 800 µM in BMMs and across a broad dosage range (0.1–300 mg), melatonin shows promise as an adjuvant therapy for managing osteoporosis and enhancing bone health [125, 134].

### 5.1 Circadian Synchronization of Melatonin

**Figure 2:**
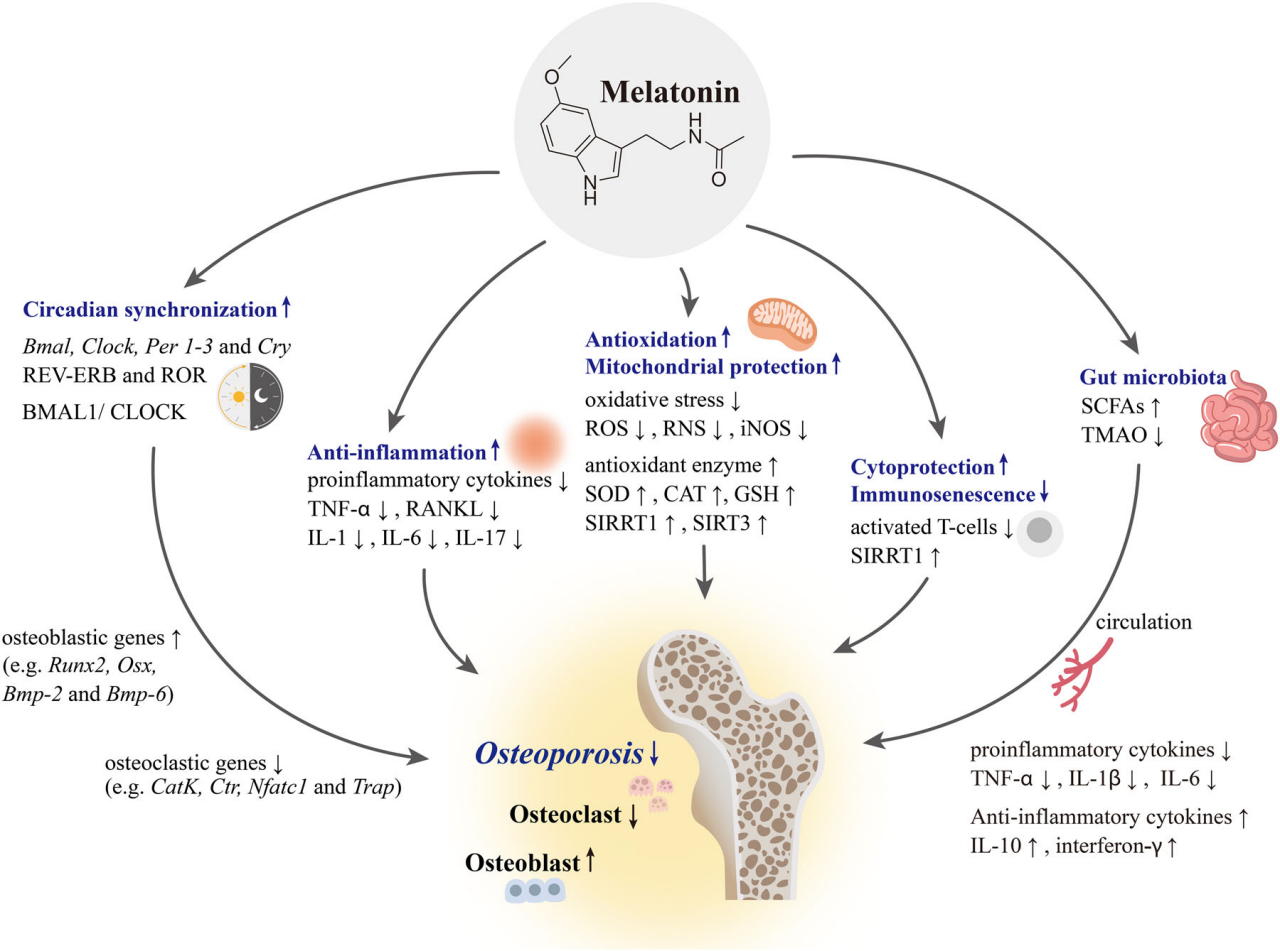
Multifaceted functions of melatonin on osteoblasts and osteoclasts in osteoporosis. Melatonin prevents osteoporosis through multifaceted actions, including modulation of circadian clock genes, anti‐inflammatory and antioxidant effects, mitochondrial protection, immunosenescence regulation, cytoprotection, and interactions within the gut–bone axis. BMAL/*Bmal*: brain and muscle aryl hydrocarbon receptor nuclear translocator‐like protein/gene; BMP/*Bmp*: bone morphogenetic protein/gene; CAT: catalase; *CatK*: cathepsin K gene; CLOCK/*Clock*: circadian locomotor output cycles kaput/gene; *Cry*: cryptochrome gene; *Ctr*: calcitonin receptor gene; GSH: glutathione; IL: interleukin; iNOS: inducible nitric oxide synthase; *Nfatc1*: nuclear factor of activated T cells gene, cytoplasmic 1; *Osx*: osterix gene; *Per*: period circadian regulator gene; RANKL: receptor activator of nuclear factor‐κ‐light‐chain enhancer of activated B cells ligand; RNS: reactive nitrogen species; REV‐ERB: nuclear receptor subfamily 1 group D member 1 (NR1D1); ROR: retinoid acid‐related orphan receptor; ROS: reactive oxygen species; *Runx2*: runt‐related transcription factor 2 gene; SCFA: short‐chain fatty acid; SIRT: silent information regulator type (Sirtuin); SOD: superoxide dismutase; TMAO: trimethylamine‐*N*‐oxide; TNF‐α: tumor necrosis factor‐α; *Trap*: tartrate‐resistant acid phosphatase gene; Wnt: Wingless‐related integration site.

The central circadian clock regulates peripheral clocks in bone cells, including osteoblasts, osteoclasts, and chondrocytes via the BMAL/circadian locomotor output cycles kaput (CLOCK) heterodimer, which activates core clock genes, including *Bmal1*, *Clock*, period (*Per*) *1–3*, and cryptochrome (*Cry*), to synchronize bone cell activities [135, 136, 137, 138] (Figure 2). Nuclear receptors REV‐ERB and ROR fine‐tune this loop by inhibiting and enhancing BMAL1 transcription, respectively [136]. Circadian rhythms, including melatonin cycles, influence expression of osteoblastic genes (e.g., *Runx2*, O*sx*, *Bmp‐2*, and *Bmp‐6*) and osteoclastic genes (e.g., *CatK* and *Nfatc1*) [137].

*Bmal1* is crucial for bone, cartilage, and tooth formation, with its deficiency linked to impaired osteoblast and chondrocyte differentiation, increased osteoclast activity, and reduced bone mass [97]. In postmenopausal women, disrupted circadian rhythms, particularly reduced *Bmal1* expression, correlate with increased bone resorption and decreased bone formation, significantly contributing to osteoporosis [94]. Melatonin, which regulates circadian rhythms, influences osteoblastic genes such as *Runx2*, *Bmp‐2* and *Bmp‐6*, and *Ocn*. These genes follow circadian patterns synchronized with light/dark cycles and melatonin levels [139]. Furthermore, melatonin enhances osteoblast differentiation through the ERK1/2‐RUNX2 signaling pathway via MT receptors [36, 69]. It also supports bone health by suppressing osteoclast activity and upregulating bone‐related genes, including *Dmp1* (dentin matrix protein), *Spp1* (OPN/*Opn*), and *Bsp* (bone sialoprotein) [106, 135, 136, 139].

Melatonin enhances *Runx2* expression and osteogenesis via NF‐κB/cyclo‐2 (COX‐2) and MAPK pathways and mitigates microgravity‐induced bone loss by modulating calcitonin receptor (*Ctr*) and *Rankl* expression through miRNAs/miRs (e.g., miR‐92b‐5p and miR‐3653‐3p) and circular RNAs (circRNAs) [90]. Additionally, it promotes osteogenesis by upregulating RUNX2, mineralization, and miR‐181c‐5p, which inhibits *Notch2* expression [95]. Its regulation of clock genes, miRNAs, and circRNAs highlights its therapeutic potential for addressing circadian disturbances and preventing bone degradation, particularly in postmenopausal osteoporosis [104].

### 5.2 Anti‐Inflammatory Effects of Melatonin

Inflammation is strongly associated with osteoporosis, particularly in chronic stages, where it suppresses osteoblast function and exacerbates bone loss and systemic bone weakening [140]. Proinflammatory cytokines such as TNF‐α, RANKL, IL‐1, IL‐6, and IL‐17 promote bone destruction by increasing osteoclast activity while inhibiting preosteoblast differentiation and mineralization through downregulation of RUNX2 and osterix, resulting in decreased production of COL1 and OCN [141]. TNF‐α further contributes to bone resorption by enhancing osteoclastic activity and inhibiting osteoblastic formation via the upregulation of SMURF1 and SMURF2, which also degrade RUNX2 [142].

Melatonin supports bone homeostasis by enhancing osteogenesis, increasing the OPG/RANKL ratio to inhibit osteoclast activity, reducing oxidative stress and inflammation, and preventing inflammation‐induced bone loss [60]. Under inflammatory conditions, it promotes Wnt4 expression through the ERK1/2‐Pax2‐Egr1 signaling pathway, enhancing the expression of osteogenic genes such as *Runx2*, osterix (*Osx*), *Alp*, and *Ocn* to stimulate bone formation [59]. Both In Vitro and In Vivo studies demonstrate their anti‐inflammatory and anti‐resorptive properties [124]. Melatonin reduces the activity of proinflammatory cytokines (e.g., TNF‐α, IL‐1, and IL‐6), modulates immune responses by inducing T‐helper (Th)2 cells and inhibiting Th1 cells, and improves insulin sensitivity, further mitigating bone loss [92, 120, 143, 144, 145]. With its dual ability to reduce bone resorption and enhance bone formation, melatonin emerges as a promising therapeutic agent for managing inflammatory bone diseases and preventing inflammation‐induced osteoporosis.

### 5.3 Antioxidant and Mitochondrial Protective Effects of Melatonin

Oxidative stress and autophagic changes are closely linked to osteoporosis, a condition that melatonin helps mitigate through its potent antioxidant properties [3]. In aging individuals and postmenopausal women, melatonin counteracts elevated ROS levels and diminished antioxidant capacity, both of which contribute to bone loss [146]. By enhancing osteoblastogenesis, suppressing osteoclastogenesis, and scavenging free radicals, melatonin improves bone density [40]. Its antioxidant properties reduce mitochondrial damage and oxidative stress by neutralizing ROS/RNS, stimulating antioxidant enzymes, and binding transition metals to prevent hydroxyl radical formation [147]. Melatonin also protects lipids, proteins, and DNA, particularly within mitochondria, from oxidative damage [148]. Notably, it outperforms vitamin E in scavenging free radicals and enhances the activity of antioxidant enzymes, including SOD, catalase (CAT), glutathione (GSH) peroxidase (GSH‐Px), and GSH reductase, while promoting intrinsic GSH synthesis [144, 149]. Furthermore, melatonin safeguards mitochondrial DNA, reduces oxidative damage, improves insulin sensitivity, and mitigates oxidative stress‐induced bone loss, reinforcing its protective role in bone health [148, 150].

Melatonin enhances OPG production, reduces osteoclast activity, and stimulates osteogenesis through AMPK, FOXO3a, and RUNX2 signaling pathways [151]. It produces antioxidant metabolites such as cyclic 3‐hydroxymelatonin (3OHM), *N*1‐acetyl‐*N*2‐formyl‐5‐methoxykynuramine (AFMK), and *N*1‐acetyl‐5‐methoxykynuramine (AMK), which help preserve mitochondrial function, prevent osteoporotic bone degradation, and induce apoptosis in osteoclast precursors [130, 152, 153, 154]. Melatonin restores MnSOD activity, maintains mitochondrial ATP production under oxidative stress from H_2_O_2_, and regulates respiratory chain complexes I and IV, preventing cytochrome *c* release and apoptosis via MT_1_ receptor‐mediated pathways [155]. Additionally, it counteracts TNF‐α‐induced ROS generation and osteogenesis inhibition, showcasing therapeutic potential in postmenopausal osteoporosis models [89, 96].

At pharmacological doses, melatonin reduces intracellular ROS induced by RANKL, suppresses osteoclastogenesis through ROS‐mediated, SIRT1‐independent mechanisms, and neutralizes oxygen‐centered radicals, thereby reducing oxidative damage critical for osteoclast differentiation [156]. It also scavenges nitrogen‐based radicals, such as NO and peroxynitrite, by inhibiting NO synthase activity, and works synergistically with other antioxidants to minimize cellular damage [157]. The strong correlation between melatonin levels and blood antioxidant capacity in humans highlights its role in mitigating oxidative stress [150, 158]. Furthermore, melatonin activates SIRT1, further suppressing osteoclastogenesis by inhibiting NF‐κB transcriptional activity [159]. In addition to acting as a potent free radical scavenger, melatonin also activates the mitochondrial MT_1_ signaling pathway, suppressing stress‐mediated cytochrome c release and caspase activation, thereby serving a dual protective role [160]. These multifaceted effects position melatonin as a promising therapeutic agent for osteoporosis associated with oxidative stress.

### 5.4 Effects of Melatonin on Immunosenescence and Cytoprotection

Melatonin is integral to immunomodulation through its interaction with high‐affinity receptors on immune cells, such as lymphocytes, which are also capable of producing melatonin [161]. These lymphocytes coordinate localized immune responses by activating both innate immunity (e.g., antigen‐presenting cells and natural killer cells) and adaptive immunity (e.g., CD4^+^ T lymphocytes) [161]. They further regulate cytokine production, stimulate cell proliferation, and inhibit apoptosis [161]. As a natural free radical scavenger and immune enhancer, melatonin protects hematopoietic cells in the bone marrow from oxidative stress while enhancing immune functions in lymphocytes [161].

A highly conserved molecule across phylogenetic lines, melatonin functions as both a chronobiotic and a cytoprotective agent [162]. However, achieving cytoprotection requires pharmacologically high doses that exceed its typical interaction with MT_1_ and MT_2_ receptors [163]. Animal studies suggest cytoprotective doses range between 40 and 100 mg/day, far surpassing the lower doses, typically under 10 mg/day, used clinically as a chronobiotic, which remain within receptor saturation levels [38, 72, 162]. Notably, such high cytoprotective doses are usually considered off‐label use.

### 5.5 Effects of Melatonin on the Brain–Gut–Bone Axis

The gut microbiota and its metabolites regulate osteoblasts, osteoclasts, and osteocytes, thereby influencing bone metabolism and the development of osteoporosis [164]. These microbial components impact nutrient absorption and blood metabolic factors, maintaining bone homeostasis through the gut–bone axis [165]. Postmenopausal estrogen deficiency exacerbates this interaction by activating the RANKL/RANK/OPG pathway and leading to compromised intestinal barrier function, increased permeability, and the release of proinflammatory cytokines such as TNF‐α and IL‐1β; this cascade enhances osteoclast activity, accelerating bone loss and aggravating osteoporosis [105]. Furthermore, the gut microbiota contributes to bone metabolism by modulating immune responses, releasing inflammatory mediators, altering immune cell populations within bone tissue, and affecting enzymatic activity, including ROS, SOD, and CAT [164].

Specific gut microbiota strains, such as *Bifidobacterium longum*, a predominant species in healthy gastrointestinal tracts, support gut function, reduce inflammation, prevent bone loss, and enhance regulatory B cell activity, thereby promoting improved bone mass [166]. In contrast, dysbiosis, characterized by disruptions in gut microbiota composition, impairs metabolic processes and contributes to bone disorders [167]. Key metabolites such as short‐chain fatty acids (SCFAs), trimethylamine‐*N*‐oxide (TMAO), and secondary bile acids are pivotal in the brain–gut–bone axis [168]. SCFAs protect against osteoporosis by repairing the intestinal barrier and inhibiting osteoclastogenesis through the downregulation of TRAF6 and NFATc1 [168]. Conversely, elevated TMAO levels exacerbate bone loss by promoting osteoclast differentiation via ROS‐dependent NF‐κB signaling [5].

The gastrointestinal tract serves as a significant source of extra‐pineal melatonin, with tissue concentrations often surpassing blood levels [39]. Melatonin enhances SCFA production, decreases TMAO‐related metabolites, restores gut barrier function, regulates the M1/M2 macrophage balance, and lowers serum levels of proinflammatory cytokines [5]. By strengthening gut barrier integrity, promoting microbiome diversity, and maintaining metabolic homeostasis, melatonin protects the intestinal tract from inflammation [5]. Additionally, it alleviates Ti nanoparticle‐induced osteolysis through the gut microbiota‐dependent butyrate/GPR109A signaling pathway [169]. Melatonin supplementation reshapes gut microbiota, modulates SCFA and TMAO metabolism, and contributes to bone health [5].

Melatonin regulates intestinal permeability by modulating tight junction proteins, including ZO‐1, occludin, and claudin‐1, while also promoting microbial diversity [170]. Additionally, melatonin activates the Nrf2 signaling pathway, enhancing antioxidative enzymes such as SOD, GSH, GSH‐Px, and CAT [150]. This activation reduces oxidative stress, protects intestinal tissues, and mitigates inflammation by decreasing proinflammatory cytokines, including IL‐6 and TNF‐α, while increasing anti‐inflammatory cytokines such as IL‐10 and interferon‐γ [150]. Melatonin further enhances calcium absorption via transcellular and paracellular pathways, partly by increasing GSH levels to counteract oxidative stress [171]. It also modulates microbiota‐driven butyrate metabolism, preventing bone loss and supporting bone health [169]. In OVX rats, melatonin improved gut metabolism, reduced gastric inflammation, and enhanced alendronate absorption [172]. Collectively, melatonin supports the gut microenvironment, reshapes microbiome composition, regulates metabolism and nutrient uptake, and helps maintain bone structure homeostasis to ameliorate osteolysis, osteoporosis, and fracture risk [5, 169].

## 6 Conclusion

Melatonin, whether administered alone or in combination with established osteoporosis treatments, has the potential to enhance therapeutic outcomes and mitigate adverse events. As an adjuvant therapy, melatonin has been investigated alongside antiresorptive agents (e.g., BPs and denosumab) and anabolic treatments (e.g., teriparatide and romosozumab), demonstrating its ability to improve BMD, trabecular microarchitecture, and bone strength in preclinical and clinical studies [3, 173]. However, its effects on sleep disorders, fall prevention, and fracture risk remain inconclusive and warrant further research [132].

Melatonin's pharmacokinetics influence its efficacy in bone health [32]. A single fast‐release bedtime dose can advance circadian rhythms by saturating MRs at pharmaceutical levels, whereas prolonged‐release formulations taken at bedtime may counteract this initial effect by inducing a phase delay during the second half of the night [174]. Timed melatonin supplementation can restore nocturnal peaks to therapeutic levels, supporting bone health in at‐risk populations, such as postmenopausal women and older adults with osteoporosis [175]. Beyond its direct osteogenic and autacoid effects, melatonin also possesses antioxidant, immunomodulatory, proapoptotic, antiproliferative, and antiangiogenic properties, positioning it as a promising complementary or alternative therapeutic agent for osteoporosis management [3, 152]. Its role in the brain–gut–bone axis further underscores its potential in bone health regulation [12, 176]. However, critical questions remain regarding the regulation of extra‐pineal melatonin production and optimal pharmacological dosing strategies [152]. Investigating the timing of melatonin administration inevitably brings to mind the potential mirror with the pulsatile PTH therapy used to stimulate bone formation in osteoporosis treatment, as opposed to the continuous PTH elevation seen in hyperparathyroidism [177]. This analogy raises intriguing possibilities: could a similarly strategic, time‐dependent approach to melatonin administration unlock new therapeutic benefits? Such a concept warrants further investigation in both research and clinical settings.

Melatonin shows promise in osteoporosis management but is limited by fall risk concerns, inconsistent dosing, and a lack of robust clinical trials [133]. Affecting 18.3% of the population (23.1% women and 11.7% men), osteoporosis requires tailored studies for postmenopausal, senile, and secondary forms, as well as for melatonin as monotherapy or adjunct [178]. Clinical doses ( < 10 mg/day) differ significantly from those used in animal studies (1–1.5 mg/kg/day), and the optimal dosing strategy, including formulation (e.g., immediate‐release vs. controlled‐release), timing (pulsatile vs. continuous), and long‐term safety, remains uncertain, despite the absence of observed toxicity in human at doses up to 100 mg in human [38, 72, 178, 179]. Its over‐the‐counter status raises concerns over quality, misuse, drug interactions, and individual variability [179]. As a nondrug supplement, melatonin lacks regulation and long‐term human data. Rigorous research and medical supervision are essential to define its safe and effective role in bone health.

## Author Contributions

**Ko‐Hsiu Lu:** conceptualization, literature search, drafting of the manuscript, critical revision and editing of the manuscript. **Yi‐Hsien Hsieh:** conceptualization, literature search, drafting of the manuscript. **Renn‐Chia Lin:** literature search, drafting of the manuscript. **Meng‐Ying Tsai:** literature search. **Shun‐Fa Yang:** conceptualization, literature search, drafting of the manuscript, critical revision and editing of the manuscript. All authors gave final approval of the version to be submitted.

## Conflicts of Interest

The authors declare no conflicts of interest.

## Data Availability

The data that support the findings of this study are available from the corresponding author upon reasonable request.

## References

[1] K. H. Lu , C. W. Lin , Y. H. Hsieh , S. C. Su , R. J. Reiter , and S. F. Yang , “New Insights Into Antimetastatic Signaling Pathways of Melatonin in Skeletomuscular Sarcoma of Childhood and Adolescence,” Cancer and Metastasis Reviews 39, no. 1 (2020): 303–320. 32086631 10.1007/s10555-020-09845-2

[2] K. H. Lu , P. W. A. Lu , E. W. H. Lu , et al., “The Potential Remedy of Melatonin on Osteoarthritis,” Journal of Pineal Research 71, no. 3 (2021): e12762. 34435392 10.1111/jpi.12762

[3] S. Maria and P. A. Witt‐Enderby , “Melatonin Effects on Bone: Potential Use for the Prevention and Treatment for Osteopenia, Osteoporosis, and Periodontal Disease and for Use in Bone‐Grafting Procedures,” Journal of Pineal Research 56, no. 2 (2014): 115–125. 24372640 10.1111/jpi.12116

[4] R. Hardeland , J. A. Madrid , D. X. Tan , and R. J. Reiter , “Melatonin, the Circadian Multioscillator System and Health: The Need for Detailed Analyses of Peripheral Melatonin Signaling,” Journal of Pineal Research 52, no. 2 (2012): 139–166. 22034907 10.1111/j.1600-079X.2011.00934.x

[5] Y. Chen , C. Yang , Z. Deng , et al., “Gut Microbially Produced Tryptophan Metabolite Melatonin Ameliorates Osteoporosis via Modulating SCFA and TMAO Metabolism,” Journal of Pineal Research 76, no. 3 (2024): e12954. 38618998 10.1111/jpi.12954

[6] Y. Xia , S. Chen , S. Zeng , et al., “Melatonin in Macrophage Biology: Current Understanding and Future Perspectives,” Journal of Pineal Research 66, no. 2 (2019): e12547. 30597604 10.1111/jpi.12547

[7] S. Maria , R. M. Samsonraj , F. Munmun , et al., “Biological Effects of Melatonin on Osteoblast/Osteoclast Cocultures, Bone, and Quality of Life: Implications of a Role for MT2 Melatonin Receptors, MEK1/2, and MEK5 in Melatonin‐Mediated Osteoblastogenesis,” Journal of Pineal Research 64, no. 3 (2018), 10.1111/jpi.12465. PMC6711668 29285799

[8] R. L. Sack , A. J. Lewy , D. L. Erb , W. M. Vollmer , and C. M. Singer , “Human Melatonin Production Decreases With Age,” Journal of Pineal Research 3, no. 4 (1986): 379–388. 3783419 10.1111/j.1600-079x.1986.tb00760.x

[9] M. D. Walker and E. Shane , “Postmenopausal Osteoporosis,” New England Journal of Medicine 389, no. 21 (2023): 1979–1991. 37991856 10.1056/NEJMcp2307353

[10] J. E. Compston , M. R. McClung , and W. D. Leslie , “Osteoporosis,” Lancet 393, no. 10169 (2019): 364–376. 30696576 10.1016/S0140-6736(18)32112-3

[11] F. Mirza and E. Canalis , “Management of Endocrine Disease: Secondary Osteoporosis: Pathophysiology and Management,” European Journal of Endocrinology 173, no. 3 (2015): R131–R151. 25971649 10.1530/EJE-15-0118 PMC4534332

[12] A. Patel , E. W. Zhou , M. O'Brien , X. Wang , and S. Zhou , “Melatonin in Neuroskeletal Biology,” Current Opinion in Pharmacology 61 (2021): 42–48. 34607253 10.1016/j.coph.2021.08.016

[13] N. Ansari and N. A. Sims , “The Cells of Bone and Their Interactions,” Handbook of Experimental Pharmacology 262 (2020): 1–25. 32006260 10.1007/164_2019_343

[14] K. H. Lu , R. C. Lin , J. S. Yang , W. E. Yang , R. J. Reiter , and S. F. Yang , “Molecular and Cellular Mechanisms of Melatonin in Osteosarcoma,” Cells 8, no. 12 (2019): 1618. 31842295 10.3390/cells8121618 PMC6952995

[15] K. Redlich and J. S. Smolen , “Inflammatory Bone Loss: Pathogenesis and Therapeutic Intervention,” Nature Reviews Drug Discovery 11, no. 3 (2012): 234–250. 22378270 10.1038/nrd3669

[16] K.‐H. Lu , S.‐I. Wang , and S.‐F. Yang , “Denosumab Withdrawal Increases Vertebral Fracture and Mortality Risk Compared With Zoledronate,” European Journal of Endocrinology (forthcoming). 10.1093/ejendo/lvaf013 39883564

[17] S. Khosla and L. C. Hofbauer , “Osteoporosis Treatment: Recent Developments and Ongoing Challenges,” Lancet Diabetes & Endocrinology 5, no. 11 (2017): 898–907. 28689769 10.1016/S2213-8587(17)30188-2 PMC5798872

[18] K. Satomura , S. Tobiume , R. Tokuyama , et al., “Melatonin at Pharmacological Doses Enhances Human Osteoblastic Differentiation In Vitro and Promotes Mouse Cortical Bone Formation In Vivo,” Journal of Pineal Research 42, no. 3 (2007): 231–239. 17349020 10.1111/j.1600-079X.2006.00410.x

[19] L. Zhou , X. Chen , J. Yan , et al., “Melatonin at Pharmacological Concentrations Suppresses Osteoclastogenesis via the Attenuation of Intracellular ROS,” Osteoporosis International 28, no. 12 (2017): 3325–3337. 28956094 10.1007/s00198-017-4127-8 PMC9841502

[20] H. Koyama , O. Nakade , Y. Takada , T. Kaku , and K. H. W. Lau , “Melatonin at Pharmacologic Doses Increases Bone Mass by Suppressing Resorption Through Down‐Regulation of the RANKL‐Mediated Osteoclast Formation and Activation,” Journal of Bone and Mineral Research 17, no. 7 (2002): 1219–1229. 12096835 10.1359/jbmr.2002.17.7.1219

[21] F. Waldhauser and M. Dietzel , “Daily and Annual Rhythms in Human Melatonin Secretion: Role in Puberty Control,” Annals of the New York Academy of Sciences 453 (1985): 205–214. 3865581 10.1111/j.1749-6632.1985.tb11811.x

[22] F. Waldhauser , G. Weiszenbacher , E. Tatzer , et al., “Alterations in Nocturnal Serum Melatonin Levels in Humans With Growth and Aging,” Journal of Clinical Endocrinology & Metabolism 66, no. 3 (1988): 648–652. 3350912 10.1210/jcem-66-3-648

[23] Y. Xie , N. Han , F. Li , et al., “Melatonin Enhances Osteoblastogenesis of Senescent Bone Marrow Stromal Cells Through NSD2‐Mediated Chromatin Remodelling,” Clinical and Translational Medicine 12, no. 2 (2022): e746. 35220680 10.1002/ctm2.746 PMC8882236

[24] F. D. Yocca and E. Friedman , “Effect of Immobilization Stress on Rat Pineal β‐Adrenergic Receptor‐Mediated Function,” Journal of Neurochemistry 42, no. 5 (1984): 1427–1432. 6142925 10.1111/j.1471-4159.1984.tb02804.x

[25] D. B. Carr , S. M. Reppert , B. Bullen , et al., “Plasma Melatonin Increases During Exercise in Women,” Journal of Clinical Endocrinology & Metabolism 53, no. 1 (1981): 224–225. 7240379 10.1210/jcem-53-1-223

[26] A. E. Smit , M. Schilperoort , and E. M. Winter , “Restoring Rhythm to Prevent Age‐Related Fractures,” Aging 14, no. 14 (2022): 5617–5619. 35859296 10.18632/aging.204192 PMC9365551

[27] C. Hassager , J. Risteli , L. Risteli , S. B. Jensen , and C. Christiansen , “Diurnal Variation in Serum Markers of Type I Collagen Synthesis and Degradation in Healthy Premenopausal Women,” Journal of Bone and Mineral Research 7, no. 11 (1992): 1307–1311. 1466255 10.1002/jbmr.5650071110

[28] G. A. Greendale , P. Witt‐Enderby , A. S. Karlamangla , et al., “Melatonin Patterns and Levels During the Human Menstrual Cycle and After Menopause,” Journal of the Endocrine Society 4, no. 11 (2020): bvaa115. 33094207 10.1210/jendso/bvaa115 PMC7566378

[29] S. Marya , A. D. Tambe , P. A. Millner , and A. I. Tsirikos , “Adolescent Idiopathic Scoliosis: A Review of Aetiological Theories of a Multifactorial Disease,” Bone & Joint Journal 104–B, no. 8 (2022): 915–921. 10.1302/0301-620X.104B8.BJJ-2021-1638.R1 35909373

[30] F. Waldhauser , M. Waldhauser , H. R. Lieberman , M. H. Deng , H. J. Lynch , and R. J. Wurtman , “Bioavailability of Oral Melatonin in Humans,” Neuroendocrinology 39, no. 4 (1984): 307–313. 6493445 10.1159/000123997

[31] X. Ma , J. R. Idle , K. W. Krausz , and F. J. Gonzalez , “Metabolism of Melatonin by Human Cytochromes P450,” Drug Metabolism and Disposition 33, no. 4 (2005): 489–494. 15616152 10.1124/dmd.104.002410

[32] N. G. Harpsøe , L. P. H. Andersen , I. Gögenur , and J. Rosenberg , “Clinical Pharmacokinetics of Melatonin: A Systematic Review,” European Journal of Clinical Pharmacology 71, no. 8 (2015): 901–909. 26008214 10.1007/s00228-015-1873-4

[33] R. L. DeMuro , A. N. Nafziger , D. E. Blask , A. M. Menhinick , and J. S. Bertino, Jr. , “The Absolute Bioavailability of Oral Melatonin,” Journal of Clinical Pharmacology 40, no. 7 (2000): 781–784. 10883420 10.1177/00912700022009422

[34] I. F. Tresguerres , F. Tamimi , H. Eimar , et al., “Melatonin Dietary Supplement as an Anti‐Aging Therapy for Age‐Related Bone Loss,” Rejuvenation Research 17, no. 4 (2014): 341–346. 24617902 10.1089/rej.2013.1542

[35] J. A. Roth , B. G. Kim , W. L. Lin , and M. I. Cho , “Melatonin Promotes Osteoblast Differentiation and Bone Formation,” Journal of Biological Chemistry 274, no. 31 (1999): 22041–22047. 10419530 10.1074/jbc.274.31.22041

[36] S. Sethi , N. M. Radio , M. P. Kotlarczyk , et al., “Determination of the Minimal Melatonin Exposure Required to Induce Osteoblast Differentiation From Human Mesenchymal Stem Cells and These Effects on Downstream Signaling Pathways,” Journal of Pineal Research 49, no. 3 (2010): 222–238. 20626586 10.1111/j.1600-079X.2010.00784.x

[37] S. Maria , M. H. Swanson , L. T. Enderby , et al., “Melatonin‐Micronutrients Osteopenia Treatment Study (MOTS): A Translational Study Assessing Melatonin, Strontium (Citrate), Vitamin D3 and Vitamin K2 (MK7) on Bone Density, Bone Marker Turnover and Health Related Quality of Life in Postmenopausal Osteopenic Women Following a One‐Year Double‐Blind RCT and on Osteoblast‐Osteoclast Co‐Cultures,” Aging 9, no. 1 (2017): 256–285. 28130552 10.18632/aging.101158 PMC5310667

[38] A. K. Amstrup , T. Sikjaer , L. Heickendorff , L. Mosekilde , and L. Rejnmark , “Melatonin Improves Bone Mineral Density at the Femoral Neck in Postmenopausal Women With Osteopenia: A Randomized Controlled Trial,” Journal of Pineal Research 59, no. 2 (2015): 221–229. 26036434 10.1111/jpi.12252

[39] S. Pandi‐Perumal , I. Trakht , V. Srinivasan , et al., “Physiological Effects of Melatonin: Role of Melatonin Receptors and Signal Transduction Pathways,” Progress in Neurobiology 85, no. 3 (2008): 335–353. 18571301 10.1016/j.pneurobio.2008.04.001

[40] F. Munmun and P. A. Witt‐Enderby , “Melatonin Effects on Bone: Implications for Use as a Therapy for Managing Bone Loss,” Journal of Pineal Research 71, no. 1 (2021): e12749. 34085304 10.1111/jpi.12749

[41] C. Luo , Q. Yang , Y. Liu , et al., “The Multiple Protective Roles and Molecular Mechanisms of Melatonin and Its Precursor N‐Acetylserotonin in Targeting Brain Injury and Liver Damage and in Maintaining Bone Health,” Free Radical Biology and Medicine 130 (2019): 215–233. 30315933 10.1016/j.freeradbiomed.2018.10.402

[42] N. M. Radio , J. S. Doctor , and P. A. Witt‐Enderby , “Melatonin Enhances Alkaline Phosphatase Activity in Differentiating Human Adult Mesenchymal Stem Cells Grown in Osteogenic Medium via MT2 Melatonin Receptors and the MEK/ERK (1/2) Signaling Cascade,” Journal of Pineal Research 40, no. 4 (2006): 332–342. 16635021 10.1111/j.1600-079X.2006.00318.x

[43] K. Sharan , K. Lewis , T. Furukawa , and V. K. Yadav , “Regulation of Bone Mass Through Pineal‐Derived Melatonin‐MT2 Receptor Pathway,” Journal of Pineal Research 63, no. 2 (2017): e12423. 28512916 10.1111/jpi.12423 PMC5575491

[44] F. Munmun , O. A. Mohiuddin , V. T. Hoang , et al., “The Role of MEK1/2 and MEK5 in Melatonin‐Mediated Actions on Osteoblastogenesis, Osteoclastogenesis, Bone Microarchitecture, Biomechanics, and Bone Formation,” Journal of Pineal Research 73, no. 2 (2022): e12814. 35674448 10.1111/jpi.12814

[45] Y. Zhou , C. Wang , J. Si , et al., “Melatonin Up‐Regulates Bone Marrow Mesenchymal Stem Cells Osteogenic Action but Suppresses Their Mediated Osteoclastogenesis via MT2‐Inactivated NF‐κB Pathway,” British Journal of Pharmacology 177, no. 9 (2020): 2106–2122. 31900938 10.1111/bph.14972 PMC7161576

[46] Y. Zhao , G. Shao , X. Liu , and Z. Li , “Assessment of the Therapeutic Potential of Melatonin for the Treatment of Osteoporosis Through a Narrative Review of Its Signaling and Preclinical and Clinical Studies,” Frontiers in Pharmacology 13 (2022): 866625. 35645810 10.3389/fphar.2022.866625 PMC9130700

[47] X. Huang , W. Chen , C. Gu , et al., “Melatonin Suppresses Bone Marrow Adiposity in Ovariectomized Rats by Rescuing the Imbalance Between Osteogenesis and Adipogenesis Through SIRT1 Activation,” Journal of Orthopaedic Translation 38 (2023): 84–97. 36381247 10.1016/j.jot.2022.10.002 PMC9619141

[48] L. Lieben , “The Circadian Clock Controls Bone Remodelling,” Nature Reviews Rheumatology 12, no. 3 (2016): 132–133. 26841687 10.1038/nrrheum.2016.10

[49] H. M. Zhang and Y. Zhang , “Melatonin: A Well‐Documented Antioxidant With Conditional Pro‐Oxidant Actions,” Journal of Pineal Research 57, no. 2 (2014): 131–146. 25060102 10.1111/jpi.12162

[50] J. Vriend and R. J. Reiter , “Melatonin Feedback on Clock Genes: A Theory Involving the Proteasome,” Journal of Pineal Research 58, no. 1 (2015): 1–11. 25369242 10.1111/jpi.12189

[51] Y. Han , Y. M. Kim , H. S. Kim , and K. Y. Lee , “Melatonin Promotes Osteoblast Differentiation by Regulating Osterix Protein Stability and Expression,” Scientific Reports 7, no. 1 (2017): 5716. 28720849 10.1038/s41598-017-06304-x PMC5515917

[52] W. Gao , M. Lin , A. Liang , et al., “Melatonin Enhances Chondrogenic Differentiation of Human Mesenchymal Stem Cells,” Journal of Pineal Research 56, no. 1 (2014): 62–70. 24117903 10.1111/jpi.12098

[53] R. Derynck and Y. E. Zhang , “Smad‐Dependent and Smad‐Independent Pathways in TGF‐β Family Signalling,” Nature 425, no. 6958 (2003): 577–584. 14534577 10.1038/nature02006

[54] K. Nakashima , X. Zhou , G. Kunkel , et al., “The Novel Zinc Finger‐Containing Transcription Factor Osterix Is Required for Osteoblast Differentiation and Bone Formation,” Cell 108, no. 1 (2002): 17–29. 11792318 10.1016/s0092-8674(01)00622-5

[55] D. L. Lacey , E. Timms , H. L. Tan , et al., “Osteoprotegerin Ligand Is a Cytokine That Regulates Osteoclast Differentiation and Activation,” Cell 93, no. 2 (1998): 165–176. 9568710 10.1016/s0092-8674(00)81569-x

[56] M. A. St Hilaire , S. A. Rahman , J. J. Gooley , P. A. Witt‐Enderby , and S. W. Lockley , “Relationship Between Melatonin and Bone Resorption Rhythms in Premenopausal Women,” Journal of Bone and Mineral Metabolism 37, no. 1 (2019): 60–71. 29318392 10.1007/s00774-017-0896-6 PMC13383753

[57] D. C. Bauer , P. Garnero , S. L. Harrison , et al., “Biochemical Markers of Bone Turnover, Hip Bone Loss, and Fracture in Older Men: The MrOS Study,” Journal of Bone and Mineral Research 24, no. 12 (2009): 2032–2038. 19453262 10.1359/JBMR.090526 PMC2791517

[58] C. H. Kim and Y. M. Yoo , “Fluid Shear Stress and Melatonin in Combination Activate Anabolic Proteins in MC3T3‐E1 Osteoblast Cells,” Journal of Pineal Research 54, no. 4 (2013): 453–461. 23397978 10.1111/jpi.12043

[59] X. Li , Z. Li , J. Wang , et al., “Wnt4 Signaling Mediates Protective Effects of Melatonin on New Bone Formation in an Inflammatory Environment,” FASEB Journal 33, no. 9 (2019): 10126–10139. 31216173 10.1096/fj.201900093RR

[60] J. Cipolla‐Neto and F. G. Amaral , “Melatonin as a Hormone: New Physiological and Clinical Insights,” Endocrine Reviews 39, no. 6 (2018): 990–1028. 30215696 10.1210/er.2018-00084

[61] N. Suzuki and A. Hattori , “Melatonin Suppresses Osteoclastic and Osteoblastic Activities in the Scales of Goldfish,” Journal of Pineal Research 33, no. 4 (2002): 253–258. 12390509 10.1034/j.1600-079x.2002.02953.x

[62] O. Nakade , H. Koyama , H. Ariji , A. Yajima , and T. Kaku , “Melatonin Stimulates Proliferation and Type I Collagen Synthesis in Human Bone Cells In Vitro,” Journal of Pineal Research 27, no. 2 (1999): 106–110. 10496146 10.1111/j.1600-079x.1999.tb00603.x

[63] G. Oktem , S. Uslu , S. H. Vatansever , H. Aktug , M. E. Yurtseven , and A. Uysal , “Evaluation of the Relationship Between Inducible Nitric Oxide Synthase (iNOS) Activity and Effects of Melatonin in Experimental Osteoporosis in the Rat,” Surgical and Radiologic Anatomy 28, no. 2 (2006): 157–162. 16362227 10.1007/s00276-005-0065-9

[64] S. Uslu , A. Uysal , G. Oktem , M. Yurtseven , T. Tanyalçin , and G. Başdemir , “Constructive Effect of Exogenous Melatonin Against Osteoporosis After Ovariectomy in Rats,” Analytical and Quantitative Cytology and Histology 29, no. 5 (2007): 317–325. 17987812

[65] M. Sanchez‐Hidalgo , Z. Lu , D. X. Tan , M. D. Maldonado , R. J. Reiter , and R. I. Gregerman , “Melatonin Inhibits Fatty Acid‐Induced Triglyceride Accumulation in ROS17/2.8 Cells: Implications for Osteoblast Differentiation and Osteoporosis,” American Journal of Physiology—Regulatory, Integrative and Comparative Physiology 292, no. 6 (2007): R2208–R2215. 17379847 10.1152/ajpregu.00013.2007

[66] A. Zaminy , I. Ragerdi Kashani , M. Barbarestani , A. Hedayatpour , R. Mahmoudi , and A. Farzaneh Nejad , “Osteogenic Differentiation of Rat Mesenchymal Stem Cells From Adipose Tissue in Comparison With Bone Marrow Mesenchymal Stem Cells: Melatonin as a Differentiation Factor,” Iranian Biomedical Journal 12, no. 3 (2008): 133–141. 18762816

[67] L. Zhang , P. Su , C. Xu , et al., “Melatonin Inhibits Adipogenesis and Enhances Osteogenesis of Human Mesenchymal Stem Cells by Suppressing PPARγ Expression and Enhancing Runx2 Expression,” Journal of Pineal Research 49, no. 4 (2010): 364–372. 20738756 10.1111/j.1600-079X.2010.00803.x

[68] L. Liu , Y. Zhu , Y. Xu , and R. J. Reiter , “Melatonin Delays Cell Proliferation by Inducing G1 and G2/M Phase Arrest in a Human Osteoblastic Cell Line hFOB 1.19,” Journal of Pineal Research 50, no. 2 (2011): 222–231. 21108658 10.1111/j.1600-079X.2010.00832.x

[69] K. H. Park , J. W. Kang , E. M. Lee , et al., “Melatonin Promotes Osteoblastic Differentiation Through the BMP/ERK/Wnt Signaling Pathways,” Journal of Pineal Research 51, no. 2 (2011): 187–194. 21470302 10.1111/j.1600-079X.2011.00875.x

[70] W. P. Clafshenkel , J. L. Rutkowski , R. N. Palchesko , et al., “A Novel Calcium Aluminate‐Melatonin Scaffold Enhances Bone Regeneration Within a Calvarial Defect,” Journal of Pineal Research 53, no. 2 (2012): 206–218. 22462771 10.1111/j.1600-079X.2012.00989.x

[71] P. A. Witt‐Enderby , J. P. Slater , N. A. Johnson , et al., “Effects on Bone by the Light/Dark Cycle and Chronic Treatment With Melatonin and/or Hormone Replacement Therapy in Intact Female Mice,” Journal of Pineal Research 53, no. 4 (2012): 374–384. 22639972 10.1111/j.1600-079X.2012.01007.x

[72] M. P. Kotlarczyk , H. C. Lassila , C. K. O'Neil , et al., “Melatonin Osteoporosis Prevention Study (MOPS): A Randomized, Double‐Blind, Placebo‐Controlled Study Examining the Effects of Melatonin on Bone Health and Quality of Life in Perimenopausal Women,” Journal of Pineal Research 52, no. 4 (2012): 414–426. 22220591 10.1111/j.1600-079X.2011.00956.x

[73] L. Zhang , J. Zhang , Y. Ling , et al., “Sustained Release of Melatonin From Poly (Lactic‐Co‐Glycolic Acid) (PLGA) Microspheres to Induce Osteogenesis of Human Mesenchymal Stem Cells In Vitro,” Journal of Pineal Research 54, no. 1 (2013): 24–32. 22712496 10.1111/j.1600-079X.2012.01016.x

[74] M. Satué , J. M. Ramis , M. del Mar Arriero , and M. Monjo , “A New Role for 5‐Methoxytryptophol on Bone Cells Function In Vitro,” Journal of Cellular Biochemistry 116, no. 4 (2015): 551–558. 25358700 10.1002/jcb.25005

[75] X. C. Xiong , Y. Zhu , R. Ge , L. F. Liu , and W. Yuan , “Effect of Melatonin on the Extracellular‐Regulated Kinase Signal Pathway Activation and Human Osteoblastic Cell Line hFOB 1.19 Proliferation,” International Journal of Molecular Sciences 16, no. 5 (2015): 10337–10353. 25961946 10.3390/ijms160510337 PMC4463649

[76] L. Zhou , X. Chen , T. Liu , et al., “Melatonin Reverses H2 O2 ‐Induced Premature Senescence in Mesenchymal Stem Cells via the SIRT1‐Dependent Pathway,” Journal of Pineal Research 59, no. 2 (2015): 190–205. 25975679 10.1111/jpi.12250 PMC4523475

[77] S. Yildirimturk , S. Batu , C. Alatli , V. Olgac , D. Firat , and Y. Sirin , “The Effects of Supplemental Melatonin Administration on the Healing of Bone Defects in Streptozotocin‐Induced Diabetic Rats,” Journal of Applied Oral Science 24, no. 3 (2016): 239–249. 27383705 10.1590/1678-775720150570 PMC5022211

[78] Z. Ping , X. Hu , L. Wang , et al., “Melatonin Attenuates Titanium Particle‐Induced Osteolysis via Activation of Wnt/β‐Catenin Signaling Pathway,” Acta Biomaterialia 51 (2017): 513–525. 28088671 10.1016/j.actbio.2017.01.034

[79] Z. M. Chu , H. B. Li , S. X. Sun , Y. C. Jiang , B. Wang , and Y. F. Dong , “Melatonin Promotes Osteoblast Differentiation of Bone Marrow Mesenchymal Stem Cells in Aged Rats,” European Review for Medical and Pharmacological Sciences 21, no. 19 (2017): 4446–4456. 29077147

[80] C. H. Kim , E. B. Jeung , and Y. M. Yoo , “Combined Fluid Shear Stress and Melatonin Enhances the ERK/Akt/mTOR Signal in Cilia‐Less MC3T3‐E1 Preosteoblast Cells,” International Journal of Molecular Sciences 19, no. 10 (2018): 2929. 30261648 10.3390/ijms19102929 PMC6213863

[81] L. P. Palin , T. O. B. Polo , F. R. S. Batista , et al., “Daily Melatonin Administration Improves Osseointegration in Pinealectomized Rats,” Journal of Applied Oral Science 26 (2018): e20170470. 29995145 10.1590/1678-7757-2017-0470 PMC6025886

[82] W. J. Bae , J. S. Park , S. K. Kang , I. K. Kwon , and E. C. Kim , “Effects of Melatonin and Its Underlying Mechanism on Ethanol‐Stimulated Senescence and Osteoclastic Differentiation in Human Periodontal Ligament Cells and Cementoblasts,” International Journal of Molecular Sciences 19, no. 6 (2018): 1742. 29895782 10.3390/ijms19061742 PMC6032161

[83] P. Dong , X. Gu , G. Zhu , M. Li , B. Ma , and Y. Zi , “Melatonin Induces Osteoblastic Differentiation of Mesenchymal Stem Cells and Promotes Fracture Healing in a Rat Model of Femoral Fracture via Neuropeptide Y/Neuropeptide Y Receptor Y1 Signaling,” Pharmacology 102, no. 5–6 (2018): 272–280. 30227410 10.1159/000492576

[84] L. Tao and Y. Zhu , “Melatonin Regulates CRE‐Dependent Gene Transcription Underlying Osteoblast Proliferation by Activating Src and PKA in Parallel,” American Journal of Translational Research 10, no. 1 (2018): 86–100. 29422996 PMC5801349

[85] Z. Wu , X. Qiu , B. Gao , et al., “Melatonin‐Mediated miR‐526b‐3p and miR‐590‐5p Upregulation Promotes Chondrogenic Differentiation of Human Mesenchymal Stem Cells,” Journal of Pineal Research 65, no. 1 (2018): e12483. 29498095 10.1111/jpi.12483

[86] X. Meng , Y. Zhu , L. Tao , S. Zhao , and S. Qiu , “miR‐590‐3p Mediates Melatonin‐Induced Cell Apoptosis by Targeting Septin 7 in the Human Osteoblast Cell Line hFOB 1.19,” Molecular Medicine Reports 17, no. 5 (2018): 7202–7208. 29568931 10.3892/mmr.2018.8729 PMC5928678

[87] A. Rafat , A. Mohammadi Roushandeh , A. Alizadeh , N. Hashemi‐Firouzi , and Z. Golipoor , “Comparison of the Melatonin Preconditioning Efficacy Between Bone Marrow and Adipose‐Derived Mesenchymal Stem Cells,” Cell Journal 20, no. 4 (2019): 450–458. 30123990 10.22074/cellj.2019.5507 PMC6099139

[88] W. Zhou , Y. Liu , J. Shen , et al., “Melatonin Increases Bone Mass Around the Prostheses of OVX Rats by Ameliorating Mitochondrial Oxidative Stress via the SIRT3/SOD2 Signaling Pathway,” Oxidative Medicine and Cellular Longevity 2019 (2019): 4019619. 31110599 10.1155/2019/4019619 PMC6487111

[89] X. Qiu , X. Wang , J. Qiu , et al., “Melatonin Rescued Reactive Oxygen Species‐Impaired Osteogenesis of Human Bone Marrow Mesenchymal Stem Cells in the Presence of Tumor Necrosis Factor‐Alpha,” Stem Cells International 2019 (2019): 6403967. 31582985 10.1155/2019/6403967 PMC6754961

[90] Y. Li , C. Feng , M. Gao , et al., “MicroRNA‐92b‐5p Modulates Melatonin‐Mediated Osteogenic Differentiation of Bone Marrow Mesenchymal Stem Cells by Targeting ICAM‐1,” Journal of Cellular and Molecular Medicine 23, no. 9 (2019): 6140–6153. 31304676 10.1111/jcmm.14490 PMC6714169

[91] S. Qiu , Z. B. Tao , L. Tao , and Y. Zhu , “Melatonin Induces Mitochondrial Apoptosis in Osteoblasts by Regulating the STIM1/Cytosolic Calcium Elevation/ERK Pathway,” Life Sciences 248 (2020): 117455. 32088216 10.1016/j.lfs.2020.117455

[92] H. Ma , X. Wang , W. Zhang , et al., “Melatonin Suppresses Ferroptosis Induced by High Glucose via Activation of the Nrf2/HO‐1 Signaling Pathway in Type 2 Diabetic Osteoporosis,” Oxidative Medicine and Cellular Longevity 2020 (2020): 9067610. 33343809 10.1155/2020/9067610 PMC7732386

[93] J. Kobayashi‐Sun , N. Suzuki , A. Hattori , M. Yamaguchi , and I. Kobayashi , “Melatonin Suppresses Both Osteoblast and Osteoclast Differentiation Through Repression of Epidermal Erk Signaling in the Zebrafish Scale,” Biochemical and Biophysical Research Communications 530, no. 4 (2020): 644–650. 32768192 10.1016/j.bbrc.2020.07.075

[94] S. T. Anderson and G. A. FitzGerald , “Sexual Dimorphism in Body Clocks,” Science 369, no. 6508 (2020): 1164–1165. 32883849 10.1126/science.abd4964

[95] H. Murodumi , H. Shigeishi , H. Kato , et al., “Melatonin‑Induced miR‑181c‑5p Enhances Osteogenic Differentiation and Mineralization of Human Jawbone‑Derived Osteoblastic Cells,” Molecular Medicine Reports 22, no. 4 (2020): 3549–3558. 32945514 10.3892/mmr.2020.11401

[96] W. Da , L. Tao , K. Wen , Z. Tao , S. Wang , and Y. Zhu , “Protective Role of Melatonin Against Postmenopausal Bone Loss via Enhancement of Citrate Secretion From Osteoblasts,” Frontiers in Pharmacology 11 (2020): 667. 32508637 10.3389/fphar.2020.00667 PMC7248328

[97] Z. Tang , T. Xu , Y. Li , W. Fei , G. Yang , and Y. Hong , “Inhibition of CRY2 by STAT3/miRNA‐7‐5p Promotes Osteoblast Differentiation Through Upregulation of CLOCK/BMAL1/P300 Expression,” Molecular Therapy—Nucleic Acids 19 (2020): 865–876. 31982773 10.1016/j.omtn.2019.12.020 PMC6994415

[98] L. Xiao , J. Lin , R. Chen , et al., “Sustained Release of Melatonin From GelMA Liposomes Reduced Osteoblast Apoptosis and Improved Implant Osseointegration in Osteoporosis,” Oxidative Medicine and Cellular Longevity 2020 (2020): 6797154. 32566094 10.1155/2020/6797154 PMC7275204

[99] J. Huang , Y. Li , L. Wang , and C. He , “Combined Effects of Low‐Frequency Pulsed Electromagnetic Field and Melatonin on Ovariectomy‐Induced Bone Loss in Mice,” Bioelectromagnetics 42, no. 8 (2021): 616–628. 34516671 10.1002/bem.22372

[100] X. Wu , S. Qiao , W. Wang , et al., “Melatonin Prevents Peri‑Implantitis via Suppression of TLR4/NF‐κB,” Acta Biomaterialia 134 (2021): 325–336. 34271168 10.1016/j.actbio.2021.07.017

[101] X. Wang , T. Chen , Z. Deng , et al., “Melatonin Promotes Bone Marrow Mesenchymal Stem Cell Osteogenic Differentiation and Prevents Osteoporosis Development Through Modulating circ_0003865 That Sponges miR‐3653‐3p,” Stem Cell Research & Therapy 12, no. 1 (2021): 150. 33632317 10.1186/s13287-021-02224-w PMC7908669

[102] H. Han , T. Tian , G. Huang , D. Li , and S. Yang , “The lncRNA H19/miR‐541‐3p/Wnt/β‐catenin Axis Plays a Vital Role in Melatonin‐Mediated Osteogenic Differentiation of Bone Marrow Mesenchymal Stem Cells,” Aging 13, no. 14 (2021): 18257–18273. 34311444 10.18632/aging.203267 PMC8351702

[103] E. Oliveira , K. Dalla‐Costa , F. França , K. Kantovitz , and D. Peruzzo , “Influence of Melatonin Associated With the Bio‐Gide® Membrane on Osteoblast Activity: An In Vitro Study,” Acta Odontológica Latinoamericana 35, no. 2 (2022): 90–97. 36260939 10.54589/aol.35/2/90 PMC10283394

[104] W. Gao , R. Li , M. Ye , et al., “The Circadian Clock Has Roles in Mesenchymal Stem Cell Fate Decision,” Stem Cell Research & Therapy 13, no. 1 (2022): 200. 35578353 10.1186/s13287-022-02878-0 PMC9109355

[105] S. Snigdha , K. Ha , P. Tsai , T. G. Dinan , J. D. Bartos , and M. Shahid , “Probiotics: Potential Novel Therapeutics for Microbiota‐Gut‐Brain Axis Dysfunction Across Gender and Lifespan,” Pharmacology & Therapeutics 231 (2022): 107978. 34492236 10.1016/j.pharmthera.2021.107978

[106] H. Guan , N. Kong , R. Tian , et al., “Melatonin Increases Bone Mass in Normal, Perimenopausal, and Postmenopausal Osteoporotic Rats via the Promotion of Osteogenesis,” Journal of Translational Medicine 20, no. 1 (2022): 132. 35296324 10.1186/s12967-022-03341-7 PMC8925213

[107] M. Li , N. Yang , L. Hao , et al., “Melatonin Inhibits the Ferroptosis Pathway in Rat Bone Marrow Mesenchymal Stem Cells by Activating the PI3K/AKT/mTOR Signaling Axis to Attenuate Steroid‐Induced Osteoporosis,” Oxidative Medicine and Cellular Longevity 2022 (2022): 8223737. 36035224 10.1155/2022/8223737 PMC9410838

[108] T. Li , H. Liu , M. Ren , Z. Zhou , W. Jiang , and M. Yang , “Daytime Administration of Melatonin Has Better Protective Effects on Bone Loss in Ovariectomized Rats,” Journal of Orthopaedic Surgery and Research 18, no. 1 (2023): 234. 36949499 10.1186/s13018-023-03695-8 PMC10035168

[109] Y. Hu , Y. Xiong , K. Zha , et al, “Melatonin Promotes BMSCs Osteoblastic Differentiation and Relieves Inflammation by Suppressing the NF‐KappaB Pathways,” Stem Cells International 2023 (2023): 7638842. 37274021 10.1155/2023/7638842 PMC10232925

[110] R. Zhao , L. Tao , S. Qiu , et al., “Melatonin Rescues Glucocorticoid‐Induced Inhibition of Osteoblast Differentiation in MC3T3‐E1 Cells via the PI3K/AKT and BMP/Smad Signalling Pathways,” Life Sciences 257 (2020): 118044. 32622944 10.1016/j.lfs.2020.118044

[111] J. H. Son , Y. C. Cho , I. Y. Sung , I. R. Kim , B. S. Park , and Y. D. Kim , “Melatonin Promotes Osteoblast Differentiation and Mineralization of MC3T3‐E1 Cells Under Hypoxic Conditions Through Activation of PKD/p38 Pathways,” Journal of Pineal Research 57, no. 4 (2014): 385–392. 25250639 10.1111/jpi.12177

[112] S. Zheng , C. Zhou , H. Yang , et al., “Melatonin Accelerates Osteoporotic Bone Defect Repair by Promoting Osteogenesis‐Angiogenesis Coupling,” Frontiers in Endocrinology 13 (2022): 826660. 35273570 10.3389/fendo.2022.826660 PMC8902312

[113] L. Xu , L. Zhang , Z. Wang , et al., “Melatonin Suppresses Estrogen Deficiency‐Induced Osteoporosis and Promotes Osteoblastogenesis by Inactivating the NLRP3 Inflammasome,” Calcified Tissue International 103, no. 4 (2018): 400–410. 29804160 10.1007/s00223-018-0428-y

[114] Y. H. Chan , K. N. Ho , Y. C. Lee , et al., “Melatonin Enhances Osteogenic Differentiation of Dental Pulp Mesenchymal Stem Cells by Regulating MAPK Pathways and Promotes the Efficiency of Bone Regeneration in Calvarial Bone Defects,” Stem Cell Research & Therapy 13, no. 1 (2022): 73. 35183254 10.1186/s13287-022-02744-z PMC8858457

[115] X. Liu , Y. Gong , K. Xiong , et al., “Melatonin Mediates Protective Effects on Inflammatory Response Induced by Interleukin‐1 Beta in Human Mesenchymal Stem Cells,” Journal of Pineal Research 55, no. 1 (2013): 14–25. 23488678 10.1111/jpi.12045

[116] S. Lee , N. H. Le , and D. Kang , “Melatonin Alleviates Oxidative Stress‐Inhibited Osteogenesis of Human Bone Marrow‐Derived Mesenchymal Stem Cells Through AMPK Activation,” International Journal of Medical Sciences 15, no. 10 (2018): 1083–1091. 30013450 10.7150/ijms.26314 PMC6036161

[117] C. Lian , Z. Wu , B. Gao , et al., “Melatonin Reversed Tumor Necrosis Factor‐Alpha‐Inhibited Osteogenesis of Human Mesenchymal Stem Cells by Stabilizing SMAD1 Protein,” Journal of Pineal Research 61, no. 3 (2016): 317–327. 27265199 10.1111/jpi.12349

[118] M. Zheng , F. Zhang , W. Fan , et al., “Suppression of Osteogenic Differentiation and Mitochondrial Function Change in Human Periodontal Ligament Stem Cells by Melatonin at Physiological Levels,” PeerJ 8 (2020): e8663. 32181054 10.7717/peerj.8663 PMC7060754

[119] M. G. Ladizesky , V. Boggio , L. E. Albornoz , P. O. Castrillón , C. Mautalen , and D. P. Cardinali , “Melatonin Increases Oestradiol‐Induced Bone Formation in Ovariectomized Rats,” Journal of Pineal Research 34, no. 2 (2003): 143–151. 12562506 10.1034/j.1600-079x.2003.00021.x

[120] W. L. Zhang , H. Z. Meng , R. F. Yang , et al., “Melatonin Suppresses Autophagy in Type 2 Diabetic Osteoporosis,” Oncotarget 7, no. 32 (2016): 52179–52194. 27438148 10.18632/oncotarget.10538 PMC5239543

[121] M. G. Ladizesky , R. A. Cutrera , V. Boggio , et al., “Effect of Melatonin on Bone Metabolism in Ovariectomized Rats,” Life Sciences 70, no. 5 (2001): 557–565. 11811900 10.1016/s0024-3205(01)01431-x

[122] Z. Ostrowska , B. Kos‐Kudla , B. Marek , et al., “The Influence of Pinealectomy and Melatonin Administration on the Dynamic Pattern of Biochemical Markers of Bone Metabolism in Experimental Osteoporosis in the Rat,” supplement, Neuro Endocrinology Letters 23 Suppl 1 (2002): 104–109. 12019362

[123] T. Histing , C. Anton , C. Scheuer , et al., “Melatonin Impairs Fracture Healing by Suppressing RANKL‐Mediated Bone Remodeling,” Journal of Surgical Research 173, no. 1 (2012): 83–90. 20888595 10.1016/j.jss.2010.08.036

[124] Z. Ping , Z. Wang , J. Shi , et al., “Inhibitory Effects of Melatonin on Titanium Particle‐Induced Inflammatory Bone Resorption and Osteoclastogenesis via Suppression of NF‐κB Signaling,” Acta Biomaterialia 62 (2017): 362–371. 28867647 10.1016/j.actbio.2017.08.046

[125] H. Kim , H. Kim , M. K. Bae , and Y. D. Kim , “Suppression of Osteoclastogenesis by Melatonin: A Melatonin Receptor‐Independent Action,” International Journal of Molecular Sciences 18, no. 6 (2017): 1142. 28587149 10.3390/ijms18061142 PMC5485966

[126] H. Jarrar , D. Çetin Altındal , and M. Gümüşderelioğlu , “Effect of melatonin/BMP‐2 Co‐Delivery Scaffolds on the Osteoclast Activity,” Journal of Materials Science: Materials in Medicine 32, no. 4 (2021): 32. 33751250 10.1007/s10856-021-06502-0 PMC7983354

[127] P. I. Liu , A. C. Chang , J. L. Lai , et al., “Melatonin Interrupts Osteoclast Functioning and Suppresses Tumor‐Secreted RANKL Expression: Implications for Bone Metastases,” Oncogene 40, no. 8 (2021): 1503–1515. 33452455 10.1038/s41388-020-01613-4

[128] J. H. Choi , A. R. Jang , M. J. Park , D. Kim , and J. H. Park , “Melatonin Inhibits Osteoclastogenesis and Bone Loss in Ovariectomized Mice by Regulating PRMT1‐Mediated Signaling,” Endocrinology 162, no. 6 (2021): bqab057. 33713122 10.1210/endocr/bqab057

[129] S. S. Kim , S. P. Jeong , B. S. Park , and I. R. Kim , “Melatonin Attenuates RANKL‐Induced Osteoclastogenesis via Inhibition of Atp6v0d2 and DC‐STAMP Through MAPK and NFATc1 Signaling Pathways,” Molecules 27, no. 2 (2022): 501. 35056817 10.3390/molecules27020501 PMC8781594

[130] X. Wang , W. Jiang , K. Pan , L. Tao , and Y. Zhu , “Melatonin Induces RAW264.7 Cell Apoptosis via the BMAL1/ROS/MAPK‐p38 Pathway to Improve Postmenopausal Osteoporosis,” Bone & Joint Research 12, no. 11 (2023): 677–690. 37907083 10.1302/2046-3758.1211.BJR-2022-0425.R3 PMC10618049

[131] A. K. Amstrup , T. Sikjaer , L. Mosekilde , and L. Rejnmark , “The Effect of Melatonin Treatment on Postural Stability, Muscle Strength, and Quality of Life and Sleep in Postmenopausal Women: A Randomized Controlled Trial,” Nutrition Journal 14 (2015): 102. 26424587 10.1186/s12937-015-0093-1 PMC4590707

[132] M. Frisher , N. Gibbons , J. Bashford , S. Chapman , and S. Weich , “Melatonin, Hypnotics and Their Association With Fracture: A Matched Cohort Study,” Age and Ageing 45, no. 6 (2016): 801–806. 27496941 10.1093/ageing/afw123

[133] T. Yang , J. Wu , X. Ding , B. Zhou , and Y. Xiong , “The Association of Melatonin Use and Hip Fracture: A Matched Cohort Study,” Osteoporosis International 34, no. 6 (2023): 1127–1135. 37036474 10.1007/s00198-023-06740-8

[134] E. J. Sanchez‐Barcelo , M. D. Mediavilla , D. X. Tan , and R. J. Reiter , “Clinical Uses of Melatonin: Evaluation of Human Trials,” Current Medicinal Chemistry 17, no. 19 (2010): 2070–2095. 20423309 10.2174/092986710791233689

[135] P. S. Welz , V. M. Zinna , A. Symeonidi , et al., “BMAL1‐Driven Tissue Clocks Respond Independently to Light to Maintain Homeostasis,” Cell 178, no. 4 (2019): 1029. 31398328 10.1016/j.cell.2019.07.030 PMC6699795

[136] F. Rijo‐Ferreira and J. S. Takahashi , “Genomics of Circadian Rhythms in Health and Disease,” Genome Medicine 11, no. 1 (2019): 82. 31847894 10.1186/s13073-019-0704-0 PMC6916512

[137] N. Huang , Y. Chelliah , Y. Shan , et al., “Crystal Structure of the Heterodimeric CLOCK:BMAL1 Transcriptional Activator Complex,” Science 337, no. 6091 (2012): 189–194. 22653727 10.1126/science.1222804 PMC3694778

[138] Z. Wang , Y. Wu , L. Li , and X. D. Su , “Intermolecular Recognition Revealed by the Complex Structure of Human CLOCK‐BMAL1 Basic Helix‐Loop‐Helix Domains With E‐Box DNA,” Cell Research 23, no. 2 (2013): 213–224. 23229515 10.1038/cr.2012.170 PMC3567813

[139] M. Ikegame , A. Hattori , M. J. Tabata , et al., “Melatonin Is a Potential Drug for the Prevention of Bone Loss During Space Flight,” Journal of Pineal Research 67, no. 3 (2019): e12594. 31286565 10.1111/jpi.12594 PMC6771646

[140] J. Chang , Z. Wang , E. Tang , et al., “Inhibition of Osteoblastic Bone Formation by Nuclear Factor‐κB,” Nature Medicine 15, no. 6 (2009): 682–689. 10.1038/nm.1954 PMC2768554 19448637

[141] H. Kaneki , R. Guo , D. Chen , et al., “Tumor Necrosis Factor Promotes Runx2 Degradation Through Up‐Regulation of Smurf1 and Smurf2 in Osteoblasts,” Journal of Biological Chemistry 281, no. 7 (2006): 4326–4333. 16373342 10.1074/jbc.M509430200 PMC2647592

[142] J. Ding , O. Ghali , P. Lencel , et al., “TNF‐α and IL‐1β Inhibit RUNX2 and Collagen Expression but Increase Alkaline Phosphatase Activity and Mineralization in Human Mesenchymal Stem Cells,” Life Sciences 84, no. 15–16 (2009): 499–504. 19302812 10.1016/j.lfs.2009.01.013

[143] A. V. Shaji , S. K. Kulkarni , and J. N. Agrewala , “Regulation of Secretion of IL‐4 and IgG1 Isotype by Melatonin‐Stimulated Ovalbumin‐Specific T Cells,” Clinical and Experimental Immunology 111, no. 1 (1998): 181–185. 9472679 10.1046/j.1365-2249.1998.00493.x PMC1904848

[144] W. Ren , G. Liu , S. Chen , et al., “Melatonin Signaling in T Cells: Functions and Applications,” Journal of Pineal Research 62, no. 3 (2017): 12394, 10.1111/jpi.12394. 28152213

[145] X. Li , C. Li , W. Zhang , Y. Wang , P. Qian , and H. Huang , “Inflammation and Aging: Signaling Pathways and Intervention Therapies,” Signal Transduction and Targeted Therapy 8, no. 1 (2023): 239. 37291105 10.1038/s41392-023-01502-8 PMC10248351

[146] S. F. Vatner , J. Zhang , M. Oydanich , T. Berkman , R. Naftalovich , and D. E. Vatner , “Healthful Aging Mediated by Inhibition of Oxidative Stress,” Ageing Research Reviews 64 (2020): 101194. 33091597 10.1016/j.arr.2020.101194 PMC7710569

[147] A. Galano , M. E. Medina , D. X. Tan , and R. J. Reiter , “Melatonin and Its Metabolites as Copper Chelating Agents and Their Role in Inhibiting Oxidative Stress: A Physicochemical Analysis,” Journal of Pineal Research 58, no. 1 (2015): 107–116. 25424557 10.1111/jpi.12196

[148] G. Paradies , V. Paradies , F. M. Ruggiero , and G. Petrosillo , “Mitochondrial Bioenergetics Decay in Aging: Beneficial Effect of Melatonin,” Cellular and Molecular Life Sciences 74, no. 21 (2017): 3897–3911. 28785806 10.1007/s00018-017-2619-5 PMC11107727

[149] T. W. Fischer , K. Kleszczyński , L. H. Hardkop , N. Kruse , and D. Zillikens , “Melatonin Enhances Antioxidative Enzyme Gene Expression (CAT, GPx, SOD), Prevents Their UVR‐Induced Depletion, and Protects Against the Formation of DNA Damage (8‐Hydroxy‐2’‐Deoxyguanosine) in Ex Vivo Human Skin,” Journal of Pineal Research 54, no. 3 (2013): 303–312. 23110400 10.1111/jpi.12018

[150] T. Yousefi , M. Yousef Memar , A. Ahmadi Jazi , et al., “Molecular Pathways and Biological Roles of Melatonin and Vitamin D; Effects on Immune System and Oxidative Stress,” International Immunopharmacology 143, no. Pt 3 (2024): 113548. 39488920 10.1016/j.intimp.2024.113548

[151] F. He , X. Liu , K. Xiong , et al., “Extracellular Matrix Modulates the Biological Effects of Melatonin in Mesenchymal Stem Cells,” Journal of Endocrinology 223, no. 2 (2014): 167–180. 25210047 10.1530/JOE-14-0430

[152] D. P. Cardinali , “Melatonin as a Chronobiotic/Cytoprotective Agent in Bone. Doses Involved,” Journal of Pineal Research 76, no. 1 (2024): e12931. 38083808 10.1111/jpi.12931

[153] S. Raha and B. H. Robinson , “Mitochondria, Oxygen Free Radicals, Disease and Ageing,” Trends in Biochemical Sciences 25, no. 10 (2000): 502–508. 11050436 10.1016/s0968-0004(00)01674-1

[154] R. J. Reiter , S. Rosales‐Corral , D. X. Tan , M. J. Jou , A. Galano , and B. Xu , “Melatonin as a Mitochondria‐Targeted Antioxidant: One of Evolution's Best Ideas,” Cellular and Molecular Life Sciences 74, no. 21 (2017): 3863–3881. 28864909 10.1007/s00018-017-2609-7 PMC11107735

[155] D. X. Tan , L. Manchester , L. Qin , and R. Reiter , “Melatonin: A Mitochondrial Targeting Molecule Involving Mitochondrial Protection and Dynamics,” International Journal of Molecular Sciences 17, no. 12 (2016): 2124. 27999288 10.3390/ijms17122124 PMC5187924

[156] J. J. García , L. López‐Pingarrón , P. Almeida‐Souza , et al., “Protective Effects of Melatonin in Reducing Oxidative Stress and in Preserving the Fluidity of Biological Membranes: A Review,” Journal of Pineal Research 56, no. 3 (2014): 225–237. 24571249 10.1111/jpi.12128

[157] H. Ebelt , D. Peschke , H. J. Brömme , W. Mörke , R. Blume , and E. Peschke , “Influence of Melatonin on Free Radical‐Induced Changes in Rat Pancreatic Beta‐Cells In Vitro,” Journal of Pineal Research 28, no. 2 (2000): 65–72. 10709967 10.1034/j.1600-079x.2001.280201.x

[158] R. J. Reiter , D. X. Tan , and M. D. Maldonado , “Melatonin as an Antioxidant: Physiology Versus Pharmacology,” Journal of Pineal Research 39, no. 2 (2005): 215–216. 16098101 10.1111/j.1600-079X.2005.00261.x

[159] M. Shakibaei , C. Buhrmann , and A. Mobasheri , “Resveratrol‐Mediated SIRT‐1 Interactions With p300 Modulate Receptor Activator of NF‐κB Ligand (RANKL) Activation of NF‐κB Signaling and Inhibit Osteoclastogenesis in Bone‐Derived Cells,” Journal of Biological Chemistry 286, no. 13 (2011): 11492–11505. 21239502 10.1074/jbc.M110.198713 PMC3064204

[160] Y. Suofu , W. Li , F. G. Jean‐Alphonse , et al., “Dual Role of Mitochondria in Producing Melatonin and Driving GPCR Signaling to Block Cytochrome c Release,” Proceedings of the National Academy of Sciences 114, no. 38 (2017): E7997–E8006. 10.1073/pnas.1705768114 PMC5617277 28874589

[161] A. Carrillo‐Vico , J. R. Calvo , P. Abreu , et al., “Evidence of Melatonin Synthesis by Human Lymphocytes and Its Physiological Significance: Possible Role as Intracrine, Autocrine, and/or Paracrine Substance,” FASEB Journal 18, no. 3 (2004): 537–539. 14715696 10.1096/fj.03-0694fje

[162] D. P. Cardinali , “Are Melatonin Doses Employed Clinically Adequate for Melatonin‐Induced Cytoprotection,” Melatonin Research 2, no. 2 (2019): 106–132.

[163] C. Venegas , J. A. García , C. Doerrier , et al., “Analysis of the Daily Changes of Melatonin Receptors in the Rat Liver,” Journal of Pineal Research 54, no. 3 (2013): 313–321. 23110416 10.1111/jpi.12019

[164] Y. W. Zhang , Y. Wu , X. F. Liu , X. Chen , and J. C. Su , “Targeting the Gut Microbiota‐Related Metabolites for Osteoporosis: The Inextricable Connection of Gut‐Bone Axis,” Ageing Research Reviews 94 (2024): 102196. 38218463 10.1016/j.arr.2024.102196

[165] Y. W. Zhang , P. R. Song , S. C. Wang , H. Liu , Z. M. Shi , and J. C. Su , “Diets Intervene Osteoporosis via Gut‐Bone Axis,” Gut Microbes 16, no. 1 (2024): 2295432. 38174650 10.1080/19490976.2023.2295432 PMC10773645

[166] L. Sapra , N. Shokeen , K. Porwal , et al., “ *Bifidobacterium longum* Ameliorates Ovariectomy‐Induced Bone Loss via Enhancing Anti‐Osteoclastogenic and Immunomodulatory Potential of Regulatory B Cells (Bregs),” Frontiers in Immunology 13 (2022): 875788. 35693779 10.3389/fimmu.2022.875788 PMC9174515

[167] J. Greenbaum , X. Lin , K. J. Su , et al., “Integration of the Human Gut Microbiome and Serum Metabolome Reveals Novel Biological Factors Involved in the Regulation of Bone Mineral Density,” Frontiers in Cellular and Infection Microbiology 12 (2022): 853499. 35372129 10.3389/fcimb.2022.853499 PMC8966780

[168] S. Lucas , Y. Omata , J. Hofmann , et al., “Short‐Chain Fatty Acids Regulate Systemic Bone Mass and Protect From Pathological Bone Loss,” Nature Communications 9, no. 1 (2018): 55. 10.1038/s41467-017-02490-4 PMC5754356 29302038

[169] Y. Wu , F. He , C. Zhang , et al., “Melatonin Alleviates Titanium Nanoparticles Induced Osteolysis via Activation of Butyrate/GPR109A Signaling Pathway,” Journal of Nanobiotechnology 19, no. 1 (2021): 170. 34092246 10.1186/s12951-021-00915-3 PMC8182936

[170] Z. Liu , H. L. Lee , J. S. Suh , et al., “The ERα/KDM6B Regulatory Axis Modulates Osteogenic Differentiation in Human Mesenchymal Stem Cells,” Bone Research 10, no. 1 (2022): 3. 34992221 10.1038/s41413-021-00171-z PMC8738748

[171] V. Areco , M. A. Rivoira , V. Rodriguez , A. M. Marchionatti , A. Carpentieri , and N. Tolosa de Talamoni , “Dietary and Pharmacological Compounds Altering Intestinal Calcium Absorption in Humans and Animals,” Nutrition Research Reviews 28, no. 2 (2015): 83–99. 26466525 10.1017/S0954422415000050

[172] E. B. Gürler , Ö. T. Çilingir‐Kaya , I. Peker Eyüboglu , et al., “Melatonin Supports Alendronate in Preserving Bone Matrix and Prevents Gastric Inflammation in Ovariectomized Rats,” Cell Biochemistry and Function 37, no. 2 (2019): 102–112. 30815905 10.1002/cbf.3379

[173] A. K. Amstrup , T. Sikjaer , L. Mosekilde , and L. Rejnmark , “Melatonin and the Skeleton,” Osteoporosis International 24, no. 12 (2013): 2919–2927. 23716040 10.1007/s00198-013-2404-8

[174] R. Hardeland , “Melatonin and the Theories of Aging: A Critical Appraisal of Melatonin's Role in Antiaging Mechanisms,” Journal of Pineal Research 55, no. 4 (2013): 325–356. 24112071 10.1111/jpi.12090

[175] S. Tordjman , S. Chokron , R. Delorme , et al., “Melatonin: Pharmacology, Functions and Therapeutic Benefits,” Current Neuropharmacology 15, no. 3 (2017): 434–443. 28503116 10.2174/1570159X14666161228122115 PMC5405617

[176] X. Lu , S. Yu , G. Chen , et al., “Insight Into the Roles of Melatonin in Bone Tissue and Bone‑Related Diseases (Review),” International Journal of Molecular Medicine 47, no. 5 (2021): 82. 33760138 10.3892/ijmm.2021.4915 PMC7979260

[177] R. L. Jilka , “Molecular and Cellular Mechanisms of the Anabolic Effect of Intermittent PTH,” Bone 40, no. 6 (2007): 1434–1446. 17517365 10.1016/j.bone.2007.03.017 PMC1995599

[178] D. P. Cardinali and R. J. Reiter , “Clinical Use of Melatonin in Osteoporosis: Expectations Still Unmet,” Osteoporosis International 35, no. 12 (2024): 2075–2076. 39422734 10.1007/s00198-024-07261-8

[179] D. P. Cardinali , G. M. Brown , and S. R. Pandi‐Perumal , “Melatonin's Benefits and Risks as a Therapy for Sleep Disturbances in the Elderly: Current Insights,” Nature and Science of Sleep 14 (2022): 1843–1855. 10.2147/NSS.S380465 PMC9578490 36267165

